# Reduced Thermal Expansion and Improved Electrochemical Performance in Pr-Substituted SrFeO_3_ as Symmetrical Electrode for Solid Oxide Fuel Cells

**DOI:** 10.1021/acsami.4c21980

**Published:** 2025-03-27

**Authors:** Abraham Sánchez-Caballero, Javier Zamudio-García, Lucía dos Santos-Gómez, Iván da Silva, Domingo Pérez-Coll, José M. Porras-Vázquez, David Marrero-López

**Affiliations:** † Dpto. de Química Inorgánica, Cristalografía y Mineralogía, 16752 Universidad de Málaga, Malaga 29071, Spain; ‡ Instituto Universitario de Materiales y Nanotecnología, IMANA, Universidad de Málaga, Campus de Teatinos, Málaga 29071, Spain; § Department of Energy Conversion and Storage, 5205 Technical University of Denmark, Fysikvej, Building 310 Kgs, Lyngby 2800, Denmark; ∥ ISIS Neutron and Muon Source, Rutherford Appleton Laboratory, Harwell Campus, Didcot OX11 0QX, U.K.; ⊥ Instituto de Cerámica y Vidrio, CSIC, Campus de Cantoblanco, Madrid 28049, Spain; # Dpto. de Física Aplicada I, Universidad de Málaga, Málaga 29071, Spain

**Keywords:** symmetrical solid oxide cell, SrFeO_3_, thermal expansion, oxygen deficiency, electrical properties

## Abstract

Advances in doping strategies have significantly improved the properties of SrFeO_3_-based electrodes. However, challenges such as high thermal expansion coefficients and limited redox stability remain critical issues that require further investigation. This study focuses on the optimization of (Sr_1–*x*_Pr_*x*_)_0.95_FeO_3–δ_ (0 < *x* ≤ 1) series, evaluating the effects of praseodymium content on thermal expansion, redox stability, and electrochemical performance for potential application as both air and fuel electrodes in symmetrical solid oxide fuel cells. Rietveld refinements of X-ray and neutron diffraction data reveal a phase transformation from tetragonal to cubic symmetry with Pr content (0.2 ≤ *x* ≤ 0.4), followed by a transition to orthorhombic symmetry (*x* ≥ 0.6). Thermogravimetric and dilatometric analyses demonstrate that higher Pr content effectively reduces both oxygen nonstoichiometry and the thermal expansion coefficients, which decrease from 31 × 10^–6^ K^–1^ for *x* = 0.2 to 8.4 × 10^–6^ K^–1^ for *x* = 1. Meanwhile the electrical conductivity remains relatively unaffected by the Pr-content up to *x* = 0.8, reaching values as high as 116 S cm^–1^ at 700 °C in air. Additionally, the electrode polarization resistances are relatively low across the series, e.g. 0.11 Ω cm^2^ in air and 0.09 Ω cm^2^ in H_2_ for *x* = 0.6 at 700 °C, while exhibiting excellent redox cycling stability. These findings indicate that (Sr_1–*x*_Pr_*x*_)_0.95_FeO_3–δ_ (*x* ≥ 0.6) materials are promising electrodes, offering tunable thermal expansion and electrochemical properties for reliable performance in both oxidizing and reducing environments.

## Graphical abstract

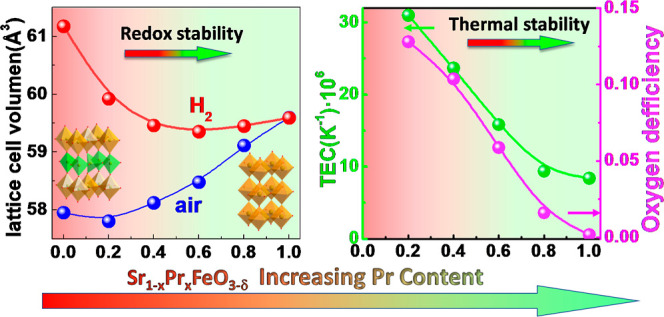

## 1 Introduction

The growing demand for cleaner and more efficient energy technologies has positioned solid oxide fuel cells (SOFCs) as a promising solution for directly converting chemical energy into electricity with minimal environmental impact.^1,2^ A typical SOFC comprises a dense electrolyte layer, commonly Zr_0.84_Y_0.16_O_1.92_ (YSZ), sandwiched between a Ni-YSZ cermet anode and a perovskite-based cathode capable of conducting both ions and electrons. However, the Ni-YSZ anode faces several challenges, including degradation under redox cycling and carbon deposition when fueled with hydrocarbons.

In recent years, symmetrical solid oxide fuel cells (SSOFCs) have emerged as a promising alternative, offering the potential to simplify the manufacturing process and lower production costs.^3,4^ This cell configuration not only reduces the complexity of assembly but also minimizes thermal expansion mismatches and prevents undesired chemical reactions between components, thus enhancing the stability and durability. Despite these advantages, the SSOFCs present several challenges that must be addressed. In particular, the electrodes need to be structurally stable, demonstrate good electrocatalytic properties, and maintain thermal compatibility with the electrolyte in both O_2_ and H_2_ environments to ensure efficient and durable operation.

La_0.75_Sr_0.25_Cr_0.5_Mn_0.5_O_3–δ_ was the first electrode successfully implemented in a SSOFC, exhibiting excellent performance with H_2_ and CH_4_ fuels at operating temperatures above 800 °C.^5^ La_1–*x*_Sr_*x*_FeO_3–δ_ has also been explored as a symmetrical electrode; however, in reducing atmosphere, it partially decomposes into multiple phases, including La_2_O_3_, which is prone to carbonation and compromises the electrode integrity.^6,7^ Nevertheless, the redox stability of these materials has been improved through appropriate B-site doping of the perovskite with cations such as Sc^3+^, Ti^4+^ or Mo^6+^.^8,9^

SrFeO_3–δ_-based materials are among the most promising electrodes for SSOFCs.^10,11^ The undoped compound SrFeO_3–δ_ crystallizes in a perovskite-type structure in air atmosphere and exhibits a relatively high electrical conductivity of 80 S cm^–1^ at 700 °C.^10,12^ However, its high thermal expansion coefficient (44 × 10^–6^ K^–1^ above 500 °C) is incompatible with common electrolytes such as YSZ (11 × 10^–6^ K^–1^).^13–15^ Moreover, under reducing conditions, SrFeO_3–δ_ undergoes a phase transition to a brownmillerite-type structure due to excessive oxygen loss caused by the reduction of Fe^4+^ to Fe^3+^.^12^ This phase transformation significantly decreases both ionic and electronic conductivity and leads to severe lattice volume changes, thereby limiting its applicability as a fuel electrode.

To address these limitations, doping the B-site of SrFeO_3–δ_ with high valence transition metals (e.g., Ti^4+^, Zr^4+^, Nb^5+^, Mo^6+^ and W^6+^) has been explored as a strategy to enhance the redox stability and prevent excessive oxygen deficiency in hydrogen atmospheres.^12,15–17^ For instance, SrFe_0.75_Zr_0.25_O_3–δ_ achieved polarization resistance values of 0.10 and 0.17 Ω cm^2^ in air and 5% H_2_, respectively, at 700 °C;^15^ however, its thermal expansion coefficients remained high (32 × 10^–6^ K^–1^). The incorporation of cations with higher oxidation states, such as Mo^6+^, successfully reduced the thermal expansion coefficient to 20.9 × 10^–6^ K^–1^ in SrFe_0.75_Mo_0.25_O_3–δ_; however, this value remains too elevated for compatibility with conventional electrolytes.^15^

A-site doping in SrFeO_3–δ_ has also been investigated by partially substituting Sr^2+^ with earth-rare elements. While La-doped SrFeO_3_ materials are unstable in reducing atmosphere, Gd-substitution enhances the redox stability.^18^ Notably, Pr-substitution offers significant advantages, improving the electronic conductivity of SrFeO_3_ via the Pr^4+^/Pr^3+^ redox pair and enhancing both the chemical and mechanical stability of the materials under both oxidizing and reducing conditions.^19,20^

In this study, we conducted a detailed structural, morphological, and electrical characterization of the (Sr_1–*x*_Pr_*x*_)_0.95_FeO_3–δ_ (0 ≤ *x* ≤ 1) series for the first time. Using X-ray and neutron powder diffraction, electron microscopy, and impedance spectroscopy, we identified the optimal composition for use as a symmetrical electrode in SSOFCs.

## 2 Experimental Section

### 2.1 Synthesis

The (Sr_1–*x*_Pr_*x*_)_0.95_FeO_3–δ_ (PSF) samples (0 ≤ *x* ≤ 1) were synthesized using a freeze-drying method, with all compositions designed to be slightly A-site deficient to minimize Sr-segregation. Pr­(NO_3_)_3_·6H_2_O (99.9%), Sr­(NO_3_)_2_ (99%) and Fe­(NO_3_)_3_·9H_2_O, (99.95%), all supplied by Merck, were dissolved in distilled water. Ethylenediaminetetraacetic acid (EDTA, 99.5% Merck) was used as a complexing agent in a 1:1 molar ratio with the metal cations. The solution was frozen dropwise into liquid nitrogen and subsequently freeze-dried for 2 days using a HyperCOOL HC3110 freeze-dryer. The resulting precursors were subjected to two consecutive thermal treatments: first at 300 °C to pyrolyze the organic components, and then at 800 °C to achieve crystallization. The powders were pressed into disks (10 mm in diameter, 1 mm in thickness) under a pressure of 100 MPa and sintered at 1100 °C for 1 h, using heating and cooling rates of 5 °C min^–1^. Postsintering, the pellets were finely ground for structural and thermal characterization. The (Sr_1–*x*_Pr_*x*_)_0.95_FeO_3–δ_ series (*x* = 0, 0.2, 0.4, 0.6, 0.8 and 1) will hereafter be referred to as Prx, where *x* indicates the praseodymium content.

### 2.2 Structural and Microstructural Characterization

The crystal structure was analyzed using X-ray powder diffraction (XRPD) with a PAN alytical X’Pert Pro MPD with CuK_α1_ radiation. Data were collected over a 2θ angular range of 10–80°, with an acquisition time of 1 h per sample. Phase identification was conducted using the X’Pert HighScore Plus software. Time-of-flight neutron powder diffraction (TOF-NPD) data were collected for selected compositions (*x* = 0.2, 0.6 and 0.8) using the GEM instrument at the ISIS pulsed spallation source (Rutherford Appleton Laboratory, UK).^21^ Approximately 6 g of powdered samples were loaded into 6 mm diameter vanadium cans, and the data were recorded at room temperature over a period of 2 h. Rietveld refinements were performed using the GSAS suite of programs.

Microstructural analysis was performed using high-angle annular dark-field scanning transmission electron microscopy (HAADF-STEM) and high-resolution transmission electron microscopy (HRTEM) on an FEI Talos F200X instrument. SEM images were acquired using a Helios Nanolab 650 equipped with energy dispersive spectroscopy (EDS, Oxford Instruments). The average grain size of the ceramics was determined from the SEM micrographs using the linear intercept method.

X-ray photoelectron spectra (XPS) data were obtained with a physical electronics PHI-5700 using MgK_α_ radiation. Binding energies were referenced to the C 1s peak at 285.0 eV. The spectra were analyzed with MultiPak software, employing Gaussian–Lorentzian functions. Full Width at Half Maximum (FWHM) values were fixed to ensure consistency in data comparison.

The thermogravimetric analysis was performed using a TA Instrument SDT Q600 in the temperature range of 25–1100 °C with heating and cooling rates of 2 °C min^–1^. The thermal expansion coefficients were determined on dense rectangular bars (12 mm in length) using a Netzsch DIL 402EP dilatometer from 25 to 800 °C in air at a heating rate of 1 °C min^–1^.

### 2.3 Electrochemical Characterization

Electrical conductivity was measured using the four-probe Van der Pauw method. Pellets (13 mm diameter, 1 mm thickness) were pressed at 200 MPa and sintered at 1100 °C for 1 h, achieving relative densities above 95%. Four platinum contacts positioned symmetrically were applied to the pellet surface for electrical connections.^22^ Conductivity measurements were performed in air and a 5% H_2_–Ar gas mixture, over a temperature range of 25–800 °C during cooling.

Symmetrical electrode cells were fabricated using La_0.9_Sr_0.1_Ga_0.8_Mg_0.2_O_3–δ_ (LSGM, KCeracell) electrolytes in the form of 10 mm diameter pellets, previously sintered at 1400 °C for 4 h. To improve electrode adhesion and reduce thermal expansion mismatch, composite electrodes were prepared by ball-milling the electrode powder with 40 wt % Ce_0.9_Gd_0.1_O_1.95_ (CGO, KCeracell) in ethanol at 150 rpm for 1 h.^23,24^ CGO, which exhibits ionic conductivity in air and mixed ionic-electronic conductivity in H_2_ atmosphere, also enhances the electrode performance. Additionally, CGO is chemically compatible with SrFeO_3_-based electrolytes at temperatures up to 1200 °C.^12^ The composite powder was mixed with Decoflux binder, screen-printed on both sides of the LSGM pellets, and sintered at 1100 °C for 1 h.

The polarization resistance of the symmetrical cells was determined using electrochemical impedance spectroscopy (EIS) with a Solartron 1260 FRA. Measurements were performed at open circuit voltage covering a frequency range from 0.01 to 10^6^ Hz, with an AC amplitude of 50 mV. The oxygen and hydrogen partial pressures were controlled using Alicat mass flow gas mixers, within a temperature range of 450–750 °C, with a 30 min dwell time between each measurement. Platinum ink and meshes were used as current collectors. EIS data were analyzed using the distribution of relaxation times (DRT) method and equivalent circuit modeling with ZView software.

Fuel cell tests were performed using a 300 μm thick LSGM symmetrical cell. The SOFCs were sealed to the alumina tube of the electrochemical setup using Ceramabond 668 ceramic paste (Aremco). Current–voltage and impedance data were recorded using a Zahner Zennium XC over a temperature range of 650–800 °C, with humidified H_2_ (3 vol % water) as the fuel at a flow rate of 20 mL min^–1^ and static air as the oxidant.

## 3 Results and Discussion

### 3.1 Structural Analysis by XRPD and NPD

The XRPD patterns of the (Sr_1–*x*_Pr_*x*_)_0.95_FeO_3–δ_ series, sintered at 1100 °C in air for 1 h, confirm that all samples are single-phases with a perovskite-type structure. However, a gradual change in symmetry is observed as the praseodymium content increases (Figure 1a). Specifically, the symmetry changes from tetragonal for SrFeO_3–δ_ (s.g. *I*4/*mmm*) to cubic (s.g. *Pm*3̅*m*) for compositions within the range of 0.2 ≤ *x* ≤ 0.4. For compositions with 0.6 ≤ *x* ≤ 1.0, the structure transitions to orthorhombic symmetry (s.g. *Pnma*). Similar trends have been reported in (Sr_1–*x*_Pr_*x*_)_0.95_FeO_3–δ_ materials synthesized using different precursor routes.^25–27^

**Figure 1:**
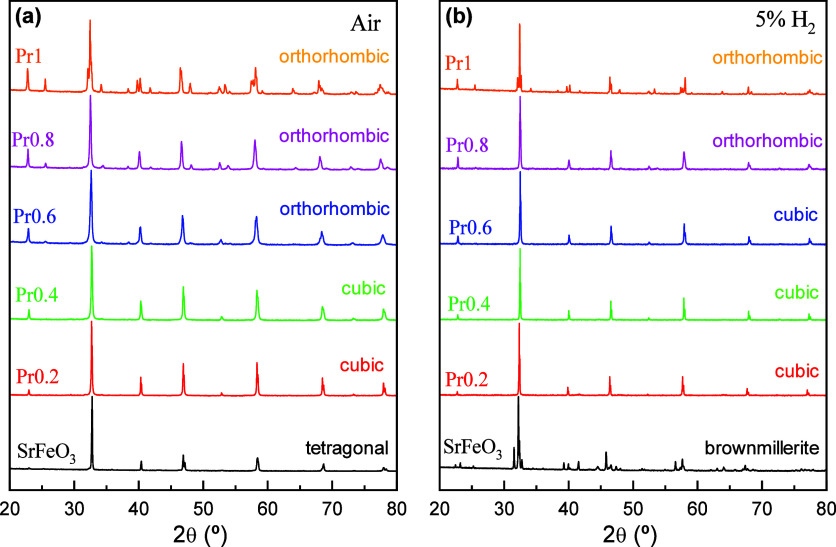
XRPD patterns of (Sr_1–*x*_Pr_*x*_)_0.95_FeO_3–δ_ (Prx) series: (a) samples synthesized in air at 1100 °C for 1 h, and (b) samples after reduction at 800 °C in 5% H_2_–Ar for 24 h.

This phase transformation can be explained by variations in the Goldsmith tolerance factor, defined as $t=\frac{r_{A}+r_{O}}{\sqrt{2}\left(r_{B}+r_{O}\right)}$ , where *r*_A_ and *r*_B_ are the ionic radii of the A-site (Sr and Pr) and B-site (Fe) cations, respectively, and *r*_O_ is the radius of oxygen. At low praseodymium content, the tolerance factor is greater than 1 due to the larger ionic radius of Sr compared to Pr, resulting in a tetragonal structure for SrFeO_3–δ_. As the Pr content increases, the effective A-site ionic radius (*r*_A_) decreases, favoring the stabilization of the cubic perovskite structure (s.g. *Pm*3̅*m*). For compositions with *x* ≥ 0.6, the tolerance factor decreases to values between 0.9 and 0.71, leading to the formation of an orthorhombic GdFeO_3_-type structure (s.g. *Pnma*).

Structural studies after annealing the samples in a 5% H_2_–Ar atmosphere at 800 °C for 24 h indicate that all samples retain their single-phase nature (Figure 1b). However, SrFeO_3–δ_ undergoes a phase transformation from a tetragonal perovskite with disordered oxygen vacancies to a brownmillerite-type structure (s.g. *Ibmm*), characterized by oxygen vacancy ordering. This transformation is attributed to partial reduction of Fe^4+^ to Fe^3+^, accompanied by a significant loss of oxide ions to maintain lattice charge neutrality.^28,29^ Such structural change, along with significant lattice expansion, limits the practical application of undoped SrFeO_3–δ_ in SSOFCs. The improved redox stability of (Sr_1–*x*_Pr_*x*_)_0.95_FeO_3–δ_ series for *x* > 0.2 is attributed to the presence of higher valence Pr ion occupying the same A-site as Sr^2+^. This substitution reduces the oxygen vacancy concentration and prevents excessive oxygen vacancy formation in reducing atmosphere, thereby stabilizing the perovskite structure.

XRPD data were analyzed using the Rietveld method, yielding satisfactory fits and good agreement factors for the proposed structural models (Figures S1 and S2 and Table S1). However, previous studies have reported discrepancies in the symmetry evolution with increasing Pr content, observing transitions through rhombohedral and tetragonal symmetries: *Pm*3̅*m → R*3̅*c → Imma → Pnma,* particularly in the range of 0.4 ≤ *x* ≤ 0.6.^25,26^ To clarify these discrepancies, neutron powder data, highly sensitive to changes in the oxygen sublattice, were collected for *x* = 0.2, 0.6, and 0.8. The results indicate excellent fitting and good agreement factors for (Sr_0.8_Pr_0.2_)_0.95_FeO_3–δ_ with a *Pm*3̅*m* space group, while (Sr_0.4_Pr_0.6_)_0.95_FeO_3–δ_ and (Sr_0.2_Pr_0.8_)_0.95_FeO_3–δ_ were refined in the *Pnma* space group, with no additional reflections associated with other symmetries (Figure 2a–c).

**Figure 2:**
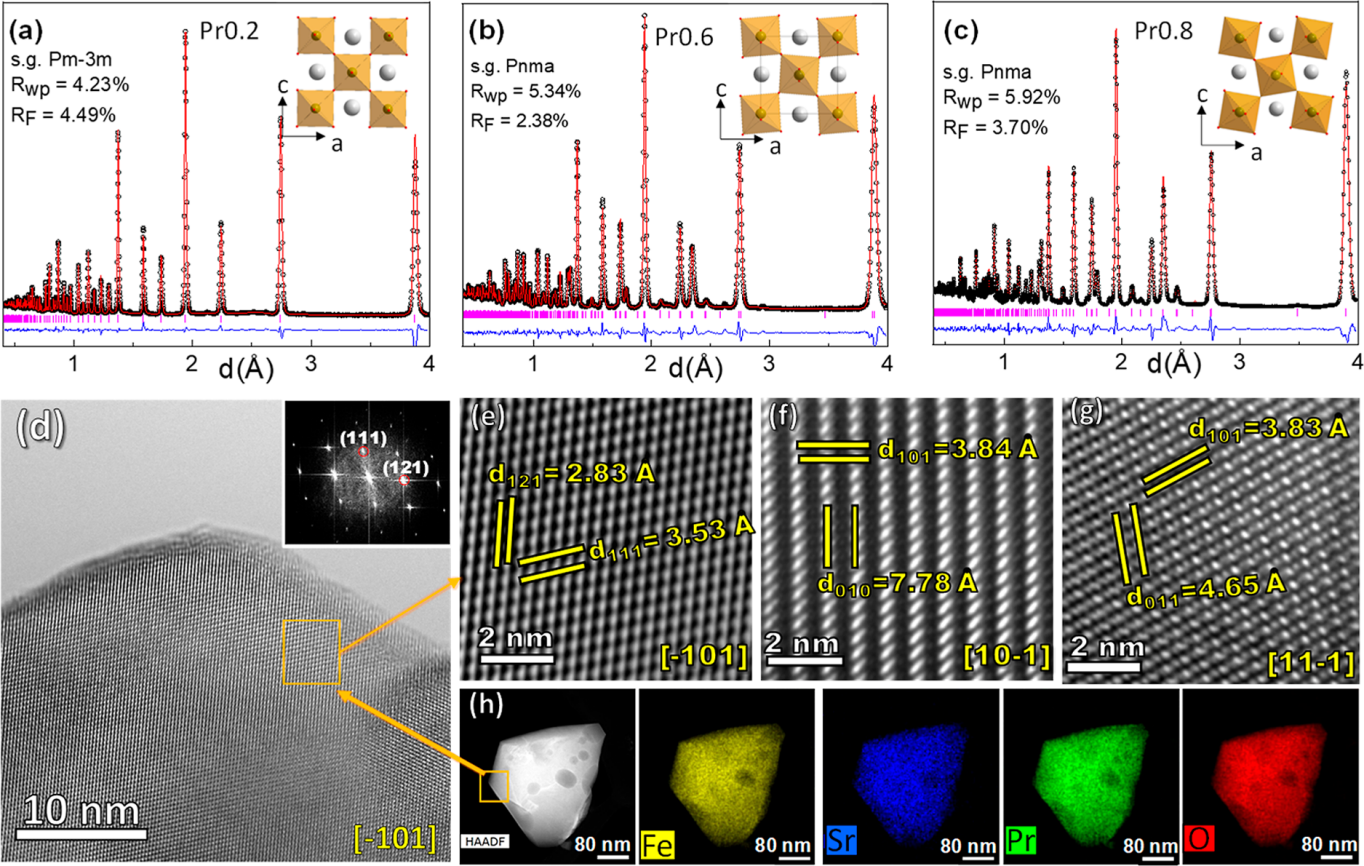
Rietveld plots of neutron powder diffraction (NPD) data for the (Sr_1–*x*_Pr_*x*_)_0.95_FeO_3–δ_ series: (a) cubic Pr0.2, (b) orthorhombic Pr0.6 and (c) orthorhombic Pr0.8. The inset shows the evolution of the octahedral tilting with increasing Pr-content. (d) Representative HRTEM image of Pr0.6 with the corresponding FFT. HRTEM images along different zone axis: (e) [−101], (f) [10–1] and (g) [11–1]. (h) HAADF-STEM of Pr0.6 with the EDS elemental distribution.

The refinement of the cationic occupancy factors is consistent with the theoretical stoichiometries (Table S2). Attempts to place praseodymium in the B-site of the perovskite, shared with iron, in an effort to explain the observed lattice volume expansion with increasing Pr content, yieded diverging refinements and poor agreement factors, confirming that Pr is located at the A-site, as initially considered. Notably, efforts to synthesize the Sr_0.95_Fe_0.9_Pr_0.1_O_3–δ_ phase led to the formation of a mixture of phases, comprising perovskite SrFeO_3–δ_ and Ruddlesden–Popper Sr_4_Fe_3_O_10_.

Meanwhile, the oxygen occupancy factors show a gradual increase in oxygen incorporation into the lattice as the Pr content increases, resulting in the following stoichiometries at room temperature: (Sr_0.8_Pr_0.2_)_0.95_FeO_2.75_, (Sr_0.4_Pr_0.6_)_0.95_FeO_2.95_ and (Sr_0.2_Pr_0.8_)_0.95_FeO_3_. As expected, the substitution of Sr^2+^ by Pr with a higher oxidation state fills the oxygen framework, reducing the number of oxide vacancies. This reduction contributes to a more stable oxygen framework, helping the material to maintain its structural integrity during redox cycling, which is crucial for achieving efficient and durable electrodes for SSOFCs. Moreover, Pr-substitution induces greater distortion in the oxygen sublattice due to the progressive tilting of the FeO_6_ octahedra, as illustrated in the insets of Figure 2a–c.

Under air conditions, the cell volume increases with increasing Pr content from 57.96 Å^3^ for SrFeO_3–δ_ to 59.55 Å^3^ for Pr1 (Figure 3). This trend might appear unexpected, as Pr^3+^ ions have a smaller ionic radius (∼1.32 Å in 12-fold coordination) compared to Sr^2+^ (1.44 Å). However, several competing factors contribute to the observed increase in cell volume. In particular, the substitution of larger Sr^2+^ ions with smaller Pr ions leads to a progressive tilting of the octahedra, which typically reduces the cell volume.^30^ However, as Pr content increases, partial reduction of Fe^4+^ to Fe^3+^ occurs, contributing to cell volume expansion. This effect is supported by the Fe–O bond distances obtained from NPD data, which increase from 1.94 Å for *x* = 0.2 to 1.99 Å for *x* = 0.8. Additionally, as Pr progressively replaces Sr, the oxygen vacancies decrease in order to maintain charge neutrality.^31^ Overall, the volume expansion observed across the (Sr_1–*x*_Pr_*x*_)_0.95_FeO_3–δ_ series is primarily governed by the reduction of Fe^4+^ ions to Fe^3+^ and the filling oxygen vacancies, which together offset the contraction effects induced by octahedral tilting and the smaller ionic size of Pr^3+^ compared to Sr^2+^.

**Figure 3:**
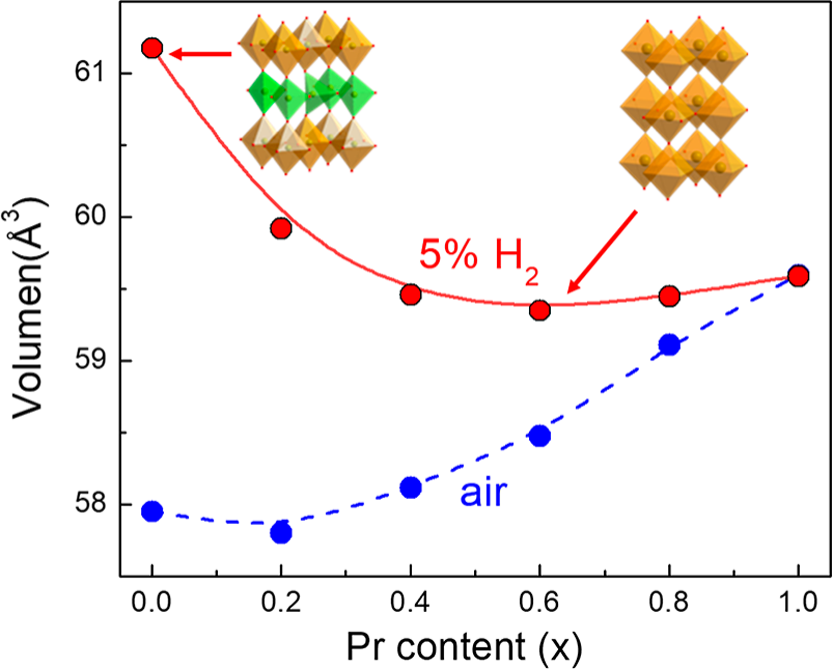
Variation of lattice cell volume of (Sr_1–*x*_Pr_*x*_)_0.95_FeO_3–δ_ series with increasing Pr content in air and 5% H_2_–Ar atmospheres.

A different trend in cell volume variation is observed when the samples are reduced in a humidified 5% H_2_–Ar atmosphere at 800 °C for 24 h. In this case, the cell volume decreases with increasing Pr content up to *x* = 0.4 and then remains nearly constant for higher Pr content (Figure 3). Interestingly, the differences in cell volume between air and hydrogen conditions progressively decrease as the Pr content increases, suggesting that the presence of Pr enhances the redox stability. This trend also indicates that oxygen nonstoichiometry, resulting from the formation of oxygen vacancies in reducing environments, plays a significant role in the observed reduction of the cell volume.

To gain a detailed understanding of the local structure of the materials, HRTEM analyses were performed. A representative HRTEM image and the corresponding fast Fourier transform (FFT) pattern for Pr0.6 are shown in Figure 2d, confirming the orthorhombic crystal structure without notable atomic defects. The interplanar distances measured along different zone axes are consistent with those determined by NPD and XRPD Rietveld refinements (Figure 2e–g). Additionally, high-angle annular dark field (HAADF) image and EDX elemental mapping confirm that Pr, Sr, Fe and O are homogeneously distributed, with no observable phase segregation (Figure 2h). This suggests that the Pr-substitution does not lead to phase separation in accordance with XRPD measurements.

### 3.2 Oxygen Deficiency and Thermal Expansion

The oxygen deficiency of the materials in air atmosphere was determined through thermogravimetric analysis (Figure 4a). The curves show a mass loss associated with oxygen release as the temperature increases. Notably, this mass loss progressively shifts to higher temperatures as the Pr content increases. The estimated oxygen deficiency (Δδ), between RT and 1100 °C in air, decreases linearly with increasing Pr content, ranging from 0.13 for *x* = 0.2 to nearly zero for *x* = 1 (inset Figure 4a).

**Figure 4:**
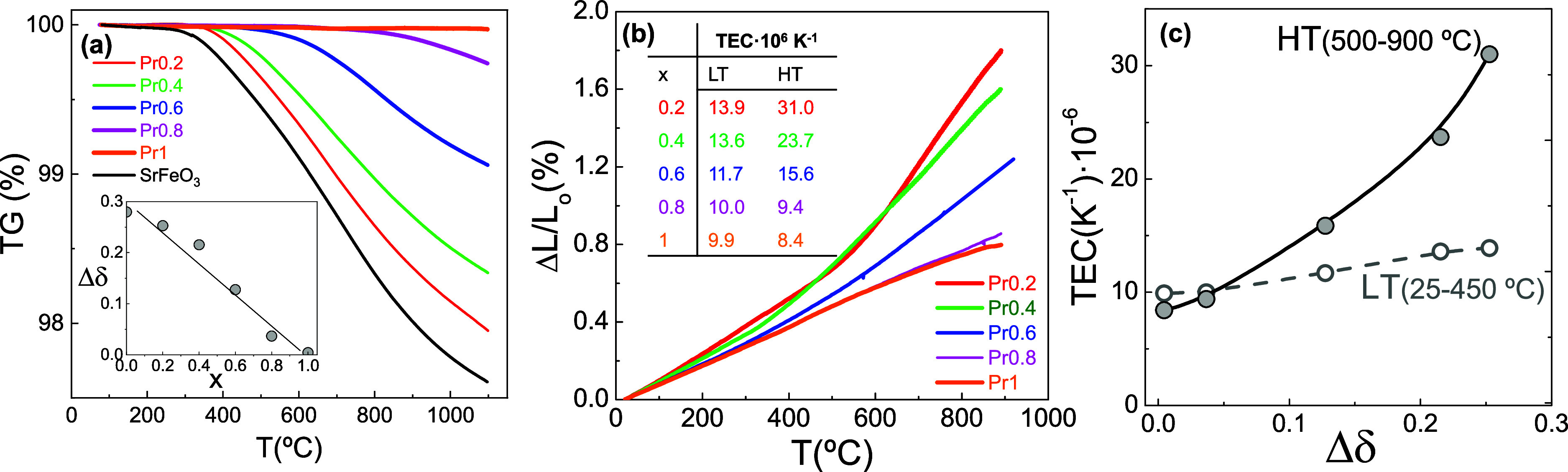
(a) Thermogravimetric and (b) dilatometric curves obtained in air for the (Sr_1–*x*_Pr_*x*_)_0.95_FeO_3–δ_ series. (c) Relationship between the oxygen deficiency (Δδ) at 1100 °C and the thermal expansion coefficients (TEC) in the high-temperature (HT) and low-temperature (LT) ranges. The inset of (a) displays the oxygen deficiency in air determined at 1100 °C.

The dilatometric curves display a trend similar to the thermogravimetric curves, showing two regions with different slopes related to oxygen release (Figure 4b). In the low-temperature (LT) region, the thermal expansion coefficients decrease with Pr content from 13.9 × 10^–6^ K^–1^ for *x* = 0.2 to 9.9 × 10^–6^ K^–1^ for *x* = 1. These differences become even more pronounced in the high-temperature (HT) region, where the thermal expansion coefficient decreases from 31 × 10^–6^ K^–1^ for *x* = 0.2 to 8.4 × 10^–6^ K^–1^ for *x* = 1. For comparison, TEC values reported in the literature for Fe-based electrodes vary in a wide range from 18.3 × 10^–6^ K^–1^ for SrFe_0.75_Mo_0.25_O_3–δ_ ^32^ to 40.15 × 10^–6^ K^–1^ for SrFe_0.9_Al_0.1_O_3–δ_ ^33^ (Table S3). Considering that typical electrolytes such as YSZ, CGO, and LSGM have thermal expansion coefficients of 10.9, 12.7 and 11.4 × 10^–6^ K^–1^, respectively, materials with *x* ≥ 0.6 are the most physically compatible to prevent potential delamination between cell layers during thermal cycling.^34^

Additionally, a clear relationship is observed between the oxygen deficiency and thermal expansion (Figure 4c). In both low-temperature (25–450 °C) and high-temperature (500–900 °C) ranges, a linear dependence is found between the thermal expansion coefficients and oxygen deficiency. These findings clearly indicate that Pr-substitution contributes to the stabilization of the oxygen sublattice, reducing the oxygen loss and mitigating thermal expansion.

### 3.3 Microstructure and Electrical Conductivity

Figure 5a–d show the SEM images of Pr0.2, Pr0.4, Pr0.6 and Pr1 pellets used for measuring the total conductivity. All ceramics have a relative density above 95%, except for Pr1, which reaches only 75% at a sintering temperature of 1100 °C. No phase segregation or inhomogeneities are observed in the ceramics. In addition, the average grain size decreases slightly with increasing Pr content up to *x* = 0.6, varying from 0.68 μm for Pr0.2 to 0.41 μm for Pr0.6. However, it then increases again for higher Pr contents, reaching 0.72 μm for Pr1. As a result, the ceramic exhibits a similar microstructure, ensuring a reliable comparison of the total conductivity within the series.

**Figure 5:**
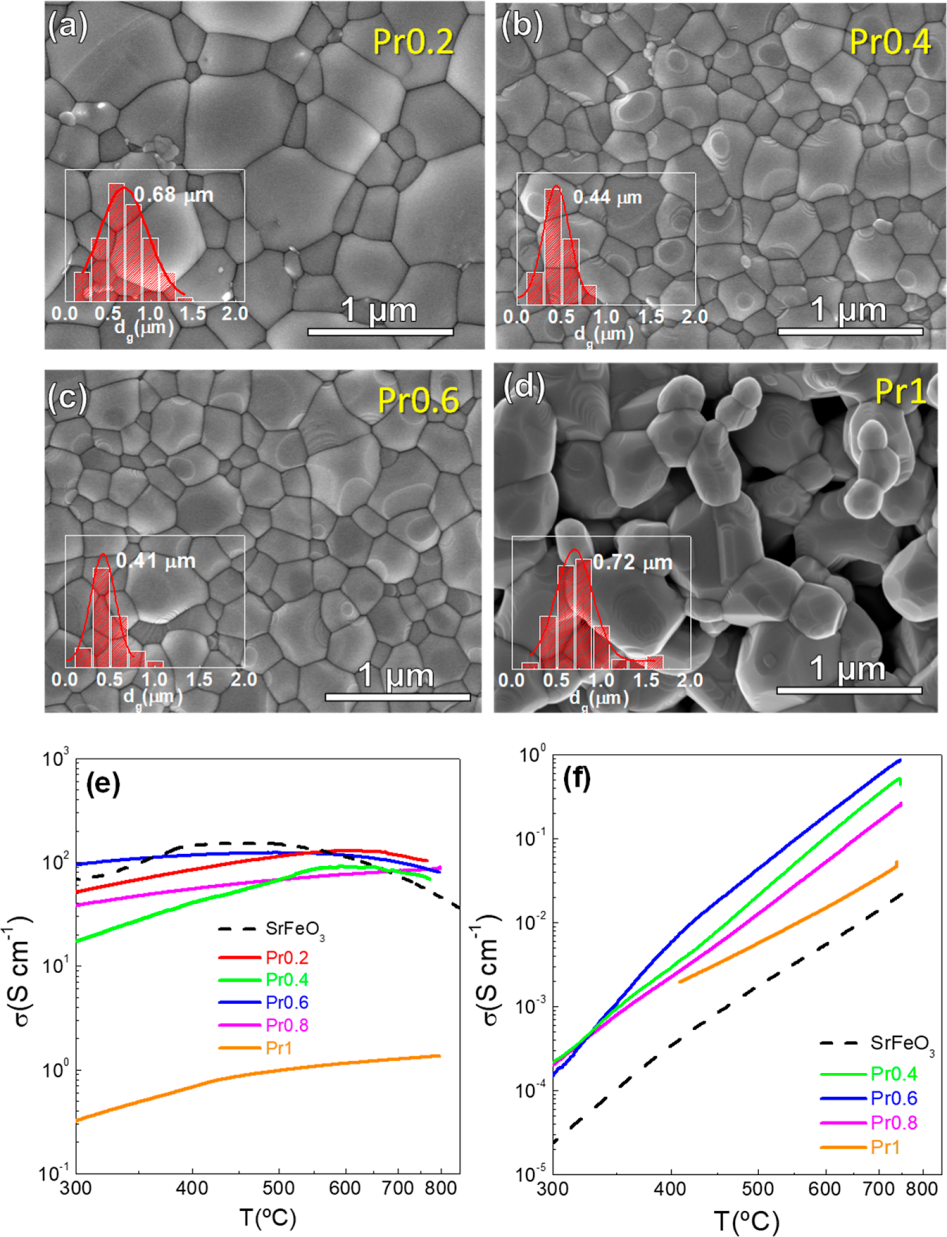
(a–d) SEM images showing the microstructure of the sintered pellets of (Sr_1–*x*_Pr_*x*_)_0.95_FeO_3–δ_ (Prx) series used for total conductivity measurements. Temperature dependence of conductivity in (e) air and (f) 5% H_2_–Ar.

The total conductivity in air displays semiconducting behavior at low temperatures, with activation energy ranging from 0.1 to 0.27 eV, indicative of polaron hopping conduction.^35^ At elevated temperatures, the conductivity decreases, likely due to thermal reduction of Fe^4+^ and the subsequent oxygen release from the lattice, which reduces the electron hole concentration, consistent with the thermogravimetric findings. A similar behavior has been reported in other Fe-based perovskites.^36–38^ In the case of undoped SrFeO_3_, the anomalous thermal behavior observed at 375 °C is attributed to a tetragonal to cubic phase transition.^12^

In the high temperature range, the different compounds exhibit similar conductivity values regardless of Pr-content, ranging from 116.2 S cm^–1^ for Pr0.2 to 99.6 S cm^–1^ for Pr0.6 at 700 °C. These values are higher compared to 74.2 S cm^–1^ for SrFeO_3_ at the same temperature. In contrast, Pr1 exhibits conductivity values 2 orders of magnitude lower (1.26 S cm^–1^ at 700 °C), attributed to a significantly lower concentration of charge carriers (Fe^4+^). This is confirmed by thermogravimetric measurements indicating a nearly stoichiometric perovskite (Figure 4a). Interestingly, samples with *x* ≤ 0.8 exhibit conductivity values that are comparable to or even higher than those reported for related compositions (Table S4), such as SrFe_0.9_Al_0.1_O_3‑δ_ (58 S cm^–1^ at 700 °C),^33^ SrFe_0.75_Zr_0.25_O_3–δ_ (8.9 S cm^–1^),^12^ La_0.8_Sr_0.2_FeO_3–δ_ (118 S cm^–1^ at 700 °C),^39^ La_0.6_Sr_0.4_Fe_0.9_Sc_0.1_O_3–δ_ (106 S cm^–1^ at 700 °C)^9^ and La_0.3_Sr_0.7_Fe_0.9_Ti_0.1_O_3–δ_ (125 S cm^–1^ at 700 °C).^40^

In a 5% H_2_–Ar atmosphere, the conductivity of all samples significantly decreases due to the partial reduction of iron to lower oxidation states, which reduces the concentration of charge carriers, primarily provided by Fe^4+^.^37,38,40^ Pr0.6 exhibits the highest conductivity, reaching 0.58 S cm^–1^ at 700 °C, a value significantly higher than that of Pr1 (0.034 S cm^–1^) and SrFeO_3_ (0.014 S cm^–1^). These values are comparable to those of other Fe-based electrodes under reducing conditions, such as La_0.8_Sr_0.2_FeO_3–δ_ (0.05 S cm^–1^ at 700 °C in H_2_)^39^ and La_0.6_Sr_0.4_Fe_0.9_Sc_0.1_O_3–δ_ (0.3 S cm^–1^ at 700 °C)^9^ (Table S4). Additionally, the observed higher activation energies in a hydrogen atmosphere, ranging between 0.60 and 0.90 eV, further evidence the hindered electronic conduction under reducing conditions.

### 3.4 XPS Analysis

The surface composition, which can differ substantially from the bulk composition but is critically important for the electrochemical reactions of the electrodes, was analyzed using XPS. The O 1s core level for representative electrodes (Pr0.2, Pr0.6 and Pr0.8) reveals four different components, in agreement with previous studies on similar Fe-based electrodes (Figure 6a).^41–43^ The most intense peak, centered at a binding energy (BE) of 528.3 eV, corresponds to lattice oxygen (O_lat_). A broader peak at 529.4 eV is attributed to surface oxygen species (O_2_^2–^/O^–^), which are related to the concentration of oxygen vacancies.^42,44^ The third peak at 531.2 eV is typically assigned to adsorbed hydroxyl groups (OH^–^/O_2_), while a minor peak at 532.7 eV corresponds to adsorbed water molecules (O_H_) (Figure 6a). As expected, the lattice oxygen increases with increasing Pr content from 40.1 at. % for Pr0.2 to 51.0 at. % for Pr0.8 (Table S5), while the concentration of oxygen vacancies decreases. This trend is consistent with findings from NPD and thermogravimetric analyses.

**Figure 6:**
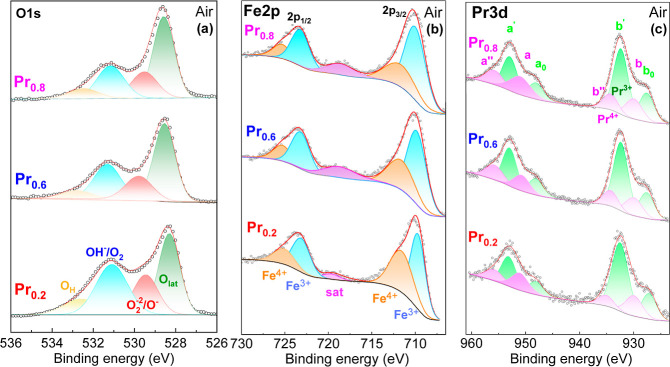
XPS core-level spectra of the surface of (Sr_1–*x*_Pr_*x*_)_0.95_FeO_3–δ_: (a) O 1s, (b) Fe 2p and (c) Pr 3d.

The deconvolution of the Fe 2p_3/2_ core level in air indicates the presence of both Fe^4+^ and Fe^3+^ species, with binding energies of 711.9 and 709.9 eV, respectively (Figure 6b). These findings are comparable to previous studies on related compositions, such as Pr_0.6_Sr_0.4_FeO_3–δ,_ ^45^ Pr_0.6_Sr_0.4_Fe_0.9_Zr_0.1_O_3–δ_ ^46^ and Pr_0.4_Sr_0.6_Fe_0.8_Co_0.2_O_3–δ_.^47^ The substitution of Sr^2+^ with Pr leads to an increase in the lattice oxygen content to maintain charge balance, while the Fe^4+^ content decreases progressively (Table S5). However, only a slight decrease in Fe^3+^ content is observed from 71.3% for Pr0.8 to 59.1% for Pr0.2, aligning with the minor variations in conductivity values in air. In general, a lower average oxidation state of Fe ions enhances the phase stability under reducing atmospheres. This improvement is achieved for Pr contents above *x* = 0.2, without significantly altering the electrical conductivity in air. Upon reduction in 5% H_2_–Ar, the Fe^3+^ signal increases, indicating further reduction of Fe^4+^ (Figure S3).^48^ This reduction in Fe^4+^ correlates with the observed decrease in conductivity under reducing atmospheres (Figure S3).

The Pr 3d core level exhibits two spin–orbit doublets, 3d_5/2_ and 3d_3/2_, revealing the presence of multiple electron satellite structures (Figure 6c). While this complicates the analysis and atomic quantification, it confirms the coexistence of both Pr^3+^ and Pr^4+^ cations on the electrode surface. The doublets labeled as (a-b, á́-b́́) correspond to Pr^4+^, while those labeled as (a_0_-b_0_, á-b́) are attributed to Pr^3+^.^49^ The characteristic peaks for Pr^4+^ (*b*) and Pr^3+^ (*b*_0_) are centered at 930.1 and 928.0 eV, respectively, which are in good agreement with values reported in the literature for similar compositions.^46,47^

### 3.5 Polarization Resistance in Symmetrical Cells

Impedance spectra under both oxidizing and reducing conditions are compared in Figure S4. In air, the compositions with intermediate Pr-content exhibit comparable polarization resistance (*R*_p_) values, despite the lower oxide vacancy concentration with increasing Pr-content: 0.14 Ω cm^2^ for Pr0.2, 0.11 Ω cm^2^ for Pr0.4 and 0.15 Ω cm^2^ for Pr0.6 at 700 °C (Figure 7). In contrast, the polarization resistance for Pr0.8 increases slightly to 0.33 Ω cm^2^, while Pr1 exhibits a significant increase to 8.75 Ω cm^2^ at the same temperature. The higher resistance of Pr1 is attributed to the low concentration of oxygen vacancies, which reduces the ionic conductivity and limits oxygen exchange. Thus, compositions with Pr content between *x* = 0.2 and *x* = 0.8 exhibit sufficient mixed ionic-electronic conductivity to achieve high oxygen reduction reaction activity. Additionally, the small particle size observed for these electrodes facilitates the formation of extended triple-phase boundary sites for the electrochemical reaction, further supporting the improved performance. These *R*_p_ values are generally lower than those reported for similar Fe-based electrodes (Table S6). For example, undoped SrFeO_3_ exhibits an *R*_p_ of 0.55 Ω cm^2^ at 700 °C, while SrFe_0.9_Mo_0.1_O_3–δ_ ^50^ achieves 0.4 Ω cm^2^ at the same temperature. These values could potentially be further reduced by employing advanced electrode deposition methods, such as spray-pyrolysis, which has demonstrated the capability to achieve an *R*_p_ of 0.1 Ω cm^2^ for both SrFe_0.9_Mo_0.1_O_3–δ_ ^50^ and Sr_0.98_Fe_0.8_Ti_0.2_O_3–δ_ at 700 °C.^51^

**Figure 7:**
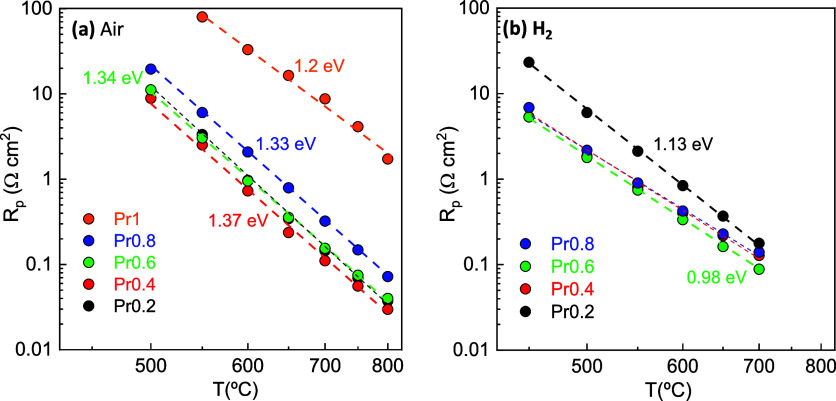
Temperature dependence of the total electrode polarization resistance in (a) air and (b) hydrogen atmospheres.

In a H_2_ atmosphere, the electrodes with high Pr-content exhibit lower *R*_p_ values, ranging from 0.09 Ω cm^2^ for Pr0.6 to 0.18 Ω cm^2^ for Pr0.2 at 700 °C. The corresponding activation energy is also comparable across different Pr contents, approximately 0.98 eV, which is lower than the values observed in air (1.20–1.37 eV), in accordance with the faster hydrogen oxidation reaction kinetics compared to oxygen reduction reaction kinetics. This enhanced performance surpasses that reported for other Fe-based electrodes, including those with high catalytic activity from exsolved metal nanoparticles (Table S6). For example, La_0.6_Ce_0.1_Sr_0.3_Fe_0.95_Ru_0.05_O_3_–CGO displays an electrode polarization resistance of 0.65 Ω cm^2^ at 700 °C.^52^ Similarly, La_0.7_Sr_0.3_Ti_0.1_Fe_0.6_Ni_0.3_O_3–δ_ has a *R*_p_ of 0.40 Ω cm^2^,^53^ while (La_0.7_Sr_0.3_)_0.8_Ti_0.1_Fe_0.6_Ni_0.3_O_3–δ_ ^54^ exhibits a value of 0.33 Ω cm^2^ at 700 °C.

The reversibility and durability of the electrodes were also evaluated by gas cycling between air and H_2_ atmospheres, consistently achieving reproducible values (Figure S6). This stable performance under varying environmental conditions highlights the robustness and redox stability of the electrodes, demonstrating their suitability for long-term application in symmetrical solid oxide fuel cells.

A comparative analysis of the electrochemical processes contributing to the electrode polarization resistance was conducted using the distribution of relaxation times (DRT) (Figure 8c,d).^55,56^ This analysis reveals two main electrode processes, regardless of the electrode composition and atmosphere. The high frequency component (HF), typically attributed to reactions at the electrode/electrolyte interface,^57,58^ is relatively low and similar for all samples, suggesting fast charge transfer and minimal interfacial reactivity between the cell layers. This finding is further supported by the comparable ohmic resistance of the cell 1.9 Ω cm^2^, similar to that of a blank LSGM electrolyte with a thickness of 1 mm. In contrast, the low frequency (LF) component, associated with surface electrochemical reactions, exhibits a higher dependence on the electrode composition under both oxidizing and reducing conditions.^58,59^

**Figure 8:**
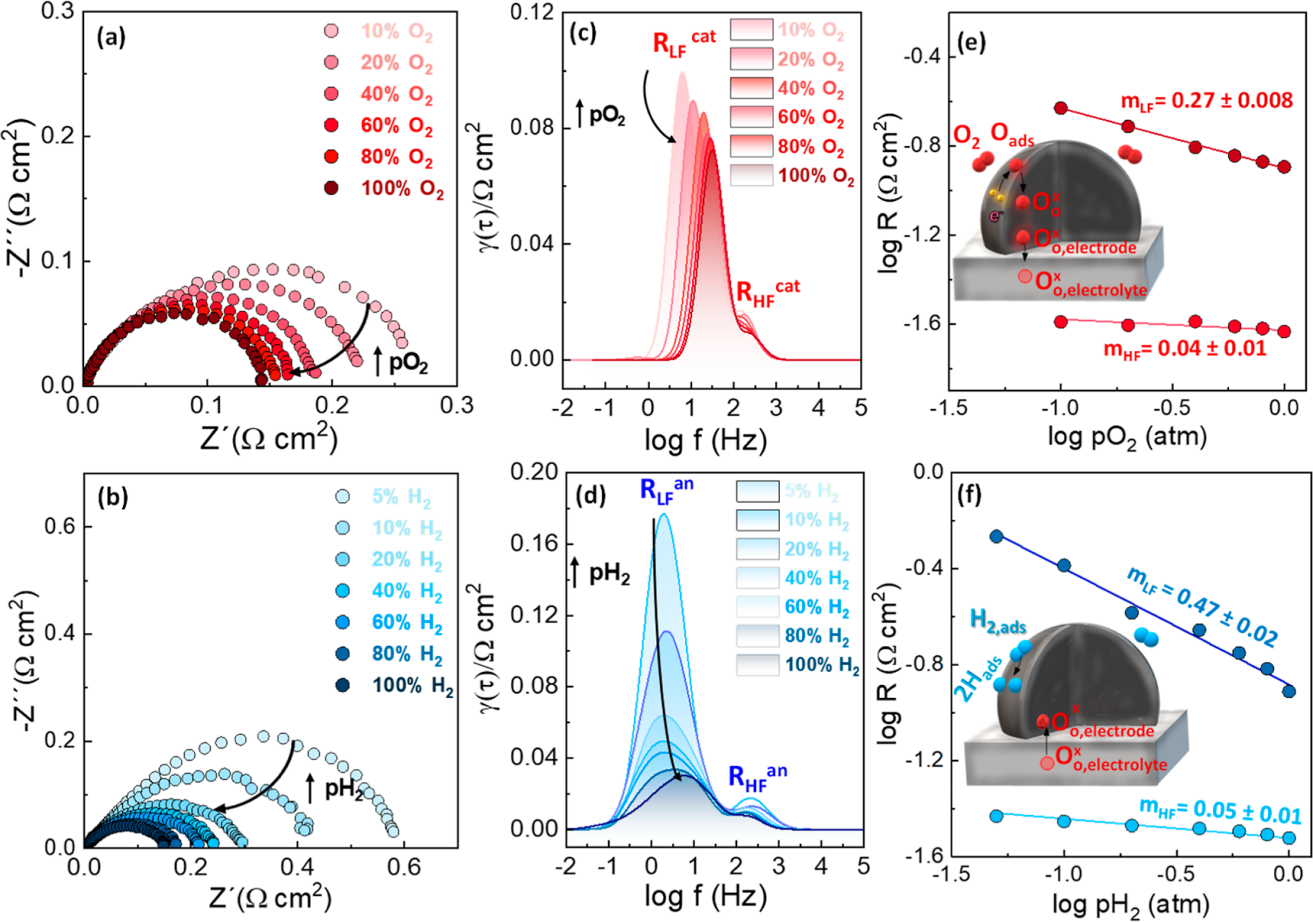
Impedance spectra at 700 °C and different (a) oxygen and (b) hydrogen partial pressures. The corresponding DRT curves (c,d) showing the main electrode contributions. Dependence of the electrode polarization resistance contributions on (e) oxygen and (f) hydrogen partial pressures. The insets figures show schematic representations of the main rate-limiting steps for the ORR and HOR.

Further insights into the nature of these electrode processes were obtained by measuring impedance spectra as a function of the oxygen partial pressure (pO_2_) and hydrogen partial pressure (pH_2_), followed by analysis using DRT curves (Figure 8). The relationship between the resistance of each specific electrode contribution and pO_2_ can be expressed as *R*_i_ ∼ (pO_2_)^−*m*^, where the value of *m* provides information about the species involved in the ORR subreactions. A similar relationship can also be established as a function of pH_2_. The high frequency (HF) response is nearly independent of both pO_2_ and pH_2_, indicating that molecular oxygen and hydrogen species are not involved in this process. Therefore, it can be attributed to the oxygen ion transport at the electrode/electrolyte interface (O^*x*^_o, electrode_ ⇌ O^*x*^_o, electrolyte_)^60^ (inset Figure 8e,f). In contrast, the low frequency (LF) contribution significantly depends on both pO_2_ and pH_2_. In oxidizing atmospheres, the LF process exhibits a reaction order of *m* = 1/4, which can be assigned to charge transfer at the electrode surface (O_ad_ + 2e^–^ + V^..^_o_ → O^*x*^_o_).^61^ Additionally, increasing pO_2_ shifts the LF process to higher frequencies, indicating a lower relaxation time and improved electrode kinetics. Under H_2_ atmosphere, the LF process exhibits a strong dependence on pH_2_, with *m* = 1/2, associated with the dissociation of adsorbed H_2_ on the electrode surface (H_2,ad_ → 2H_ad_).^62^ Similar rate limiting steps were previously observed in related electrodes, such as Zr and Ti-doped SrFeO_3_.^15,51^

### 3.6 Fuel Cell Test

The performance of an electrolyte-supported cell (300 μm thick LSGM) with Pr0.6 symmetrical electrode was studied under real operation conditions. Figure 9a presents the *I*–*V* and power density curves of the Pr0.6/LSGM/Pr0.6 symmetrical cell in static air and wet hydrogen at different temperatures. The open circuit voltage (OCV) reached 1.1 V, which is close to the theoretical Nernst potential, indicating effective sealing of the cell. The maximum power density achieved was 630, 370, and 210 mW cm^–2^ at 800, 750, and 700 °C, respectively. This performance is comparable to previously reported values for related symmetrical electrodes (Table S6), such as Sr_2_Fe_1.5_Mo_0.5_O_6–δ_ (650 mW cm^–2^ at 850 °C),^63^ La_0.5_Sr_0.5_Fe_0.9_Mo_0.1_O_3–δ_ (450 mW cm^–2^ at 800 °C)^64^ and La_0.7_Sr_0.3_Fe_0.7_Ga_0.3_O_3−δ_ (450 mW cm^–2^ at 800 °C).^65^ Additionally, these values surpass those of electrodes incorporating exsolved metal nanoparticles, such as La_0.6_Sr_0.4_Fe_0.95_Pd_0.05_O_3−δ_-SDC (350 mW cm^–2^ at 750 °C)^7^ and La_0.7_Sr_0.3_Ti_0.1_Fe_0.6_Ni_0.3_O_3–δ_ (402 mW cm^–2^ at 800 °C)^53^ (Table S6).

**Figure 9:**
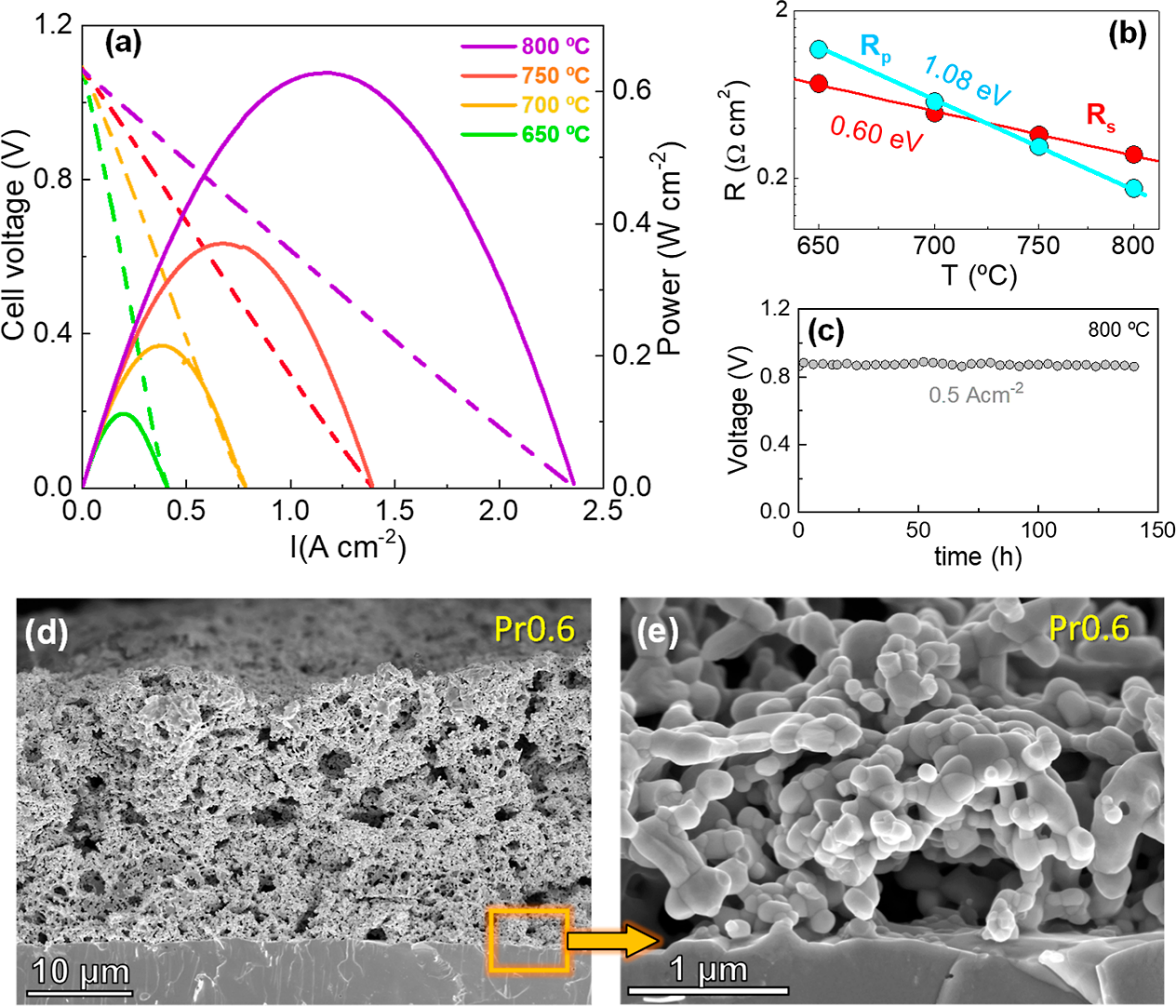
(a) *I*–*V* and *I*–*P* curves of the symmetrical cell with Pr0.6 electrode on 300 μm-thick LSGM electrolyte. (b) Temperature dependence of the total polarization resistance (*R*_p_) and series resistance (*R*_s_) of the cell. (c) Durability test of the cell at 800 °C under a constant current of 0.5 A cm^–2^. (d,e) Morphology of the electrodes after fuel cell tests.

The ohmic resistance (*R*_s_) of the cell decreased from 2.4 to 0.30 Ω cm^2^ in the temperature range of 650–800 °C, consistent with the expected values for a 300 μm thick LSGM electrolyte (Figure 9b). As the operating temperature decreases, the electrochemical activity of the electrode is reduced, leading to higher polarization resistance (*R*_p_) values that range from 0.17 Ω cm^2^ at 800 °C to 1.2 Ω cm^2^ at 650 °C. The activation energy for *R*_p_ was determined to be 1.08 eV, indicating a stronger temperature dependence compared to *R*_s_, which has a lower activation energy of 0.60 eV, similar to that of the LSGM electrolyte in the high temperature range.^66^

To further investigate the electrode processes, *I*–*V* curves and impedance spectra were recorded at different fuel concentrations at 700 °C (Figure S6a). The results indicate that the cell performance is only significantly affected when the H_2_ concentration is below 60%, with power density values of 210 mW cm^–2^ for 100% H_2_ and 180 mW cm^–2^ for 60% H_2_. Moreover, the *I*–*V* curves show a linear dependence at high current densities within this fuel concentration range. However, substantial gas transport losses become evident for fuel content below 50%, negatively affecting the overall cell performance.^67,68^

The DRT analysis of the impedance spectra shows multiple contributions (Figure S6b). The high frequency (HF) processes, attributed to the oxide ion transport at the electrode/electrolyte interfaces, remain practically unaffected by changes in fuel concentration. In contrast, the low frequency (LF) contribution, associated with electrochemical reactions at the electrode surface, becomes the dominant factor in the overall cell resistance. This LF process shifts slightly to lower frequencies, indicating slower fuel oxidation kinetics. Additionally, a third contribution appears at a very low frequency of 0.5 Hz, likely related to fuel diffusion limitations (D), as previously reported.^55,56,59^ This diffusion-related process becomes significant only when the fuel concentration is below 20%, indicating that the electrodes present optimal microstructural porosity, which facilitates adequate gas transport to the reaction sites.

The stability test of the cell at 800 °C demonstrates minimal voltage variation under a constant current density (Figure 9c). A cross-section SEM image of the cell shows a 20 μm thick electrode with high porosity and no detectable reactivity or delamination at the electrode electrolyte/interface after redox cycling (Figure 9d,e). Furthermore, the average particle size of the electrodes remains below 200 nm, indicating a stable microstructure that supports the overall integrity of the cell during prolonged operation.

In summary, although B-site doping with high valence cations, such as Ti^4+^, Nb^5+^, Mo^6+^ or W^6+^, improves the redox stability of SrFeO_3–δ_ based electrodes, it often leads to a significant reduction in both the electrical conductivity and oxygen reduction reaction activity in air.^12^ In contrast, this study demonstrates that A-site substitution with Pr in SrFeO_3‑δ_ not only maintains high conductivity levels in air but also significantly enhances ORR activity. Additionally, this approach improves the phase stability in H_2_ and results in lower thermal expansion coefficients compared to B-site doping, making these electrodes more compatible with standard electrolytes. This strategy also addresses a key limitation of conventional redox-stable electrodes used in symmetrical solid oxide cells, such as (La,Sr)­CrO_3–δ_ or (La,Sr)­TiO_3–δ_, which generally exhibit relatively poor electrochemical performance under oxidizing conditions.^69^

Thus, by balancing redox stability, conductivity and thermal expansion, Pr-substituted SrFeO_3–δ_ offers clear advantages over previously studied symmetrical electrodes. Moreover, the performance of these electrodes could be further optimized by incorporating small amounts of high valence transition metal dopants, such as Mo^6+^ or W^6+^, in the B-site of the perovskite to increase the electrical conductivity in H_2_ atmosphere. Furthermore, alternative preparation techniques, such as spray-pyrolysis deposition, could be used, as they have proven effective in enhancing the performance of conventional electrodes at lower operating temperatures.^70^

## 4 Conclusions

The effects of Pr incorporation on the structural, thermal and electrical properties of (Sr_1–*x*_Pr_*x*_)_0.95_FeO_3–δ_ (0 ≤ *x* ≤ 1) series were comprehensively investigated using different characterization techniques. A gradual phase transition was observed, starting from tetragonal symmetry for *x* = 0, evolving to cubic for 0.2 ≤ *x* ≤ 0.4, and finally to orthorhombic for *x* ≥ 0.6, accompanied by the rotation of FeO_6_ octahedra. Despite the smaller ionic radius of Pr compared to Sr, the lattice cell volume expands with increasing Pr content. This expansion, which enhances oxide ion mobility, suggests that lattice oxygen primarily influences the observed cell volume behavior. Additionally, the oxygen deficiency decreases with increasing Pr-content in both air and H_2_ atmosphere. Notably, the thermal expansion coefficients decrease significantly from 31 × 10^–6^ K^–1^ for *x* = 0.2 to 8.4 × 10^–6^ K^–1^ for *x* = 1, indicating enhanced mechanical compatibility with common electrolytes. The total electrical conductivity in air remains comparable across the series, ranging from 116.2 S cm^–1^ for Pr0.2 to 99.6 S cm^–1^ for Pr0.6 at 700 °C. However, the conductivity decreases significantly under reducing conditions, with Pr0.6 achieving the highest value of 0.58 S cm^–1^ at 700 °C in a 5% H_2_–Ar atmosphere, almost 2 orders of magnitude higher than undoped SrFeO_3–δ_. The polarization resistance in air is also similar for all compositions with Pr content between *x* = 0.2 and *x* = 0.8, despite the reduction in oxide vacancy concentration as Pr content increases, with values ranging from 0.11 to 0.33 Ω cm^2^ at 700 °C in air. In H_2_, the polarization resistance improves from 0.18 Ω cm^2^ for Pr0.2 to 0.09 Ω cm^2^ for Pr0.6_._ Thus, Pr0.6 was identified as the optimal composition, exhibiting moderate thermal expansion coefficients, improved electrical properties and stability under redox cycling, with a stable power density of 630 mW in symmetrical cell configuration cm^–2^ at 800 °C.

Overall, these findings underscore the potential of Sr_1–*x*_Pr_*x*_FeO_3–δ_ materials for symmetrical solid oxide fuel cell applications, highlighting their robust performance and stability under oxidizing and reducing conditions. Future research could focus on further enhancing the properties, particularly in H_2_ environments, through compositional modifications and microstructural optimization via alternative preparation methods at reduced deposition temperatures.

## Supplementary Material



## Notes

Tables with Lattice parameters from XRPD; structural parameters from NPD; XPS analysis; electrical conductivity; Thermal expansion and polarization resistance of Fe-based electrodes; Rietveld plots of XRPD patterns in air and H_2_; Lattice cell volume variation with Pr content; XPS spectra in H_2_ for Pr0.6; Impedance spectra and DRT curves in air and H_2_; Polarization resistance of Pr0.6 after cycling in air and H_2_; *I*–*V* and DRT curves at different fuel concentrations (PDF)
