# Supplementary material for: Reduced Thermal Expansion and Improved Electrochemical Performance in Pr-Substituted SrFeO3 as Symmetrical Electrode for Solid Oxide Fuel Cells
Source: ACS Appl Mater Interfaces. 2025 Mar 27;17(14):21380–91. doi: 10.1021/acsami.4c21980 (PMC12128030; doi:10.1021/acsami.4c21980)
Supplement: Supplementary file 1 [file am4c21980_si_001.pdf]

Supporting Information

Reduced thermal expansion and improved electrochemical performance in Pr-substituted $SrFeO_3$ as symmetrical electrode for Solid Oxide Fuel Cells

Abraham Sánchez-Caballero[1,2], *Javier Zamudio-García*[3], *Lucía dos Santos-Gómez*[1,2], *Iván da Silva*[4], *Domingo Pérez-Coll*[5], *José M. Porras-Vázquez*[1,2,*], *David Marrero-López*[2,6,*]

[1] Universidad de Málaga, Dpto. de Química Inorgánica, Cristalografía y Mineralogía, 29071-Málaga, Spain.

[2] Instituto Universitario de Materiales y Nanotecnología, IMANA, Universidad de Málaga, Campus de Teatinos 29071, Málaga, Spain

[3] Department of Energy Conversion and Storage, Technical University of Denmark, Fysikvej, Building 310, 2800 Kgs., Lyngby, Denmark.

[4] ISIS Neutron and Muon Source, Rutherford Appleton Laboratory, Harwell Campus, OX11 0QX, Didcot, UK.

[5] Instituto de Cerámica y Vidrio, CSIC, Campus de Cantoblanco, 28049 Madrid, Spain.

[6] Universidad de Málaga, Dpto. de Física Aplicada I, 29071-Málaga, Spain.

Corresponding Author

David Marrero-López - Dpto. de Física Aplicada I, Facultad de Ciencias, Campus de Teatinos, Universidad de Málaga, 29071-Málaga, Spain. Email: *marrero@uma.es*

José Manuel Porras-Vázquez - Dpto. de Química Inorgánica, Facultad de Ciencias, Campus de Teatinos, Universidad de Málaga, 29071-Málaga, Spain. Email: *josema@uma.es*

Table S1. Lattice parameters of $(Sr_{1-x}Pr_x)_{0.95}FeO_{3-\delta}$ series obtained by Rietveld refinement of XRPD data.

	x=0.2		x=0.4		x=0.6		x=0.8		x=1	
	**air**	**H_2**	**air**	**H_2**	**air**	**H_2**	**air**	**H_2**	**air**	**H_2**
**s.g.**	$Pm\bar{3}m$	$Pm\bar{3}m$	$Pm\bar{3}m$	$Pm\bar{3}m$	*Pnma*	$Pm\bar{3}m$	*Pnma*	*Pnma*	*Pnma*	*Pnma*
**a (Å)**	3.8746(1)	3.9132(1)	3.8770(1)	3.9032(1)	5.5015(3)	3.9008(1)	5.5187(2)	5.5303(3)	5.4857(1)	5.4864(1)
**b (Å)**	-	-	-	-	7.7584(2)	-	7.7804(2)	7.8057(4)	5.5736(1)	5.5753(2)
**c (Å)**	-	-	-	-	5.4833(2)	-	5.5011(2)	5.5162(3)	7.7905(2)	7.7926(2)
**V/Z (A^3)**	58.16(1)	59.92(1)	58.27(1)	59.46(1)	58.51(1)	59.35(1)	59.05(1)	59.53(1)	59.55(1)	59.59(1)
**R_{wp} (%)**	2.77	5.77	2.38	6.47	2.79	6.70	2.59	7.27	2.71	3.56
**R_F(%)**	1.52	4.60	2.58	3.20	5.40	3.02	5.91	8.01	2.70	2.60

Table S2. Structural parameters obtained by Rietveld refinement of NPD data.

	**$(Sr_{0.8}Pr_{0.2})_{0.95}FeO_{3-\delta}$**	**$(Sr_{0.4}Pr_{0.6})_{0.95}FeO_{3-\delta}$**	**$(Sr_{0.2}Pr_{0.8})_{0.95}FeO_{3-\delta}$**
**s.g.**	$Pm\bar{3}m$	*Pnma*	*Pnma*
**a (Å)**	3.8746(1)	5.4755(5)	5.5107(8)
**b (Å)**	-	7.7476(9)	7.7840(9)
**c (Å)**	-	5.5063(6)	5.5019(8)
**V/Z (A^3)**	58.17(1)	58.40(1)	59.00(1)
**R_{wp} (%)**	4.23	5.34	6.13
**R_F(%)**	4.49	2.83	3.70
**d Fe-O (Å)**	1.94	1.96	1.98
**Oxygen deficiency (δ)**	0.25	0.05	0
**Pr/Sr**			
x	0.0000	0.4905(9)	0.4746(7)
y	0.0000	0.2500	0.2500
z	0.0000	-0.0101(9)	-0.0121(9)
**U_{iso} × 100 ($Å^2$)**	0.80(2)	0.48(5)	0.58(4)
**Occ. Factor (Pr/Sr)**	0.19/0.76	0.57/0.38	0.76/0.19
**Fe**			
x	0.5000	0.0000	0.0000
y	0.5000	0.0000	0.0000
z	0.5000	0.0000	0.0000
**U_{iso} × 100 ($Å^2$)**	0.51(1)	0.37(3)	0.50(3)
**Occ. Factor**	1.00	1.00	1.00
**O(1)**			
x	0.5000	0.0010(3)	0.0055(9)
y	0.5000	0.2500	0.2500
z	0.0000	0.0772(7)	0.0834(9)
**U_{iso} × 100 ($Å^2$)**	2.07(2)	1.05(9)	1.55(9)
**Occ. factor**	0.917(1)	1.00	1.00
**O(2)**			
x		0.2643(7)	0.2785(5)
y		-0.0244(4)	-0.0437(4)

z	0.2293(6)	0.2222(7)
**U_{iso} × 100 (Å^2)**	0.93(6)	0.73(5)
**Occ. factor**	0.977(5)	1.00

Table S3. Thermal expansion coefficients of Fe-based electrodes in the low temperature (LT) and high temperature (HT) range.

**Composition**	TEC·10^6 K^{-1}		**Ref.**
	**LT**	**HT**	
Pr0.2	13.9	31.0	This work
Pr0.4	13.6	23.7	This work
Pr0.6	11.7	15.6	This work
Pr0.8	10.0	9.4	This work
Pr1	9.9	8.4	This work
$SrFeO_{3-\delta}$	18	44.2	1
$SrFe_{0.9}Zr_{0.1}O_{3--\delta}$	18.5	42.5	1
$SrFe_{0.75}Zr_{0.25}O_{3--\delta}$	14.1	32.1	1
$SrFe_{0.9}Al_{0.1}O_{3-\delta}$	18.7	40.1	2
$SrFe_{0.8}Al_{0.2}O_{3-\delta}$	14.9	30.4	2
$SrFe_{0.9}Sb_{0.1}O_{3-\delta}$	18.6	42.3	3
$SrFe_{0.95}Sb_{0.05}O_{3-\delta}$	10.6	34.5	4
$SrFe_{0.9}Mo_{0.1}O_{3-\delta}$	14.6	30.8	5
$SrFe_{0.75}Mo_{0.25}O_{3-\delta}$	13.5	18.3	6
$Sr_2Fe_{1.5}Mo_{0.5}O_{3-\delta}$	13.3	19.6	7
$SrFe_{0.8}W_{0.1}Ti_{0.1}O_{3-\delta}$	12.5		8
$SrFe_{0.8}W_{0.1}Ta_{0.1}O_{3-\delta}$	17.4	25.5	9
$SrFe_{0.5}Co_{0.4}Nb_{0.1}O_{3-\delta}$	16.1	24.4	10
$SrFe_{0.5}Ti_{0.2}Co_{0.2}Mn_{0.1}O_{3-\delta}$	16.1	27.1	11
$Sr_{0.9}Fe_{0.8}Sc_{0.1}Co_{0.1}O_{3-\delta}$	16.9	30	12
$PrBaFe_2O_{6-\delta}$	13.6	23.4	13

Table S4. Electrical conductivity of Fe-based electrodes typically used as symmetrical electrodes.

Material	σ(S cm^{-1}) (air)	σ(S cm^{-1}) (H_2)	T(ºC)	Ref.
$Sr_{0.95}FeO_{3-\delta}$	77	0.014	700	This work
$(Sr_{0.8}Pr_{0.2})_{0.95}FeO_{3-\delta}$	116	-	700	This work
$(Sr_{0.6}Pr_{0.4})_{0.95}FeO_{3-\delta}$	80.8	0.34	700	This work
$(Sr_{0.4}Pr_{0.6})_{0.95}FeO_{3-\delta}$	99.6	0.58	700	This work
$(Sr_{0.2}Pr_{0.8})_{0.95}FeO_{3-\delta}$	82.0	0.16	700	This work
$Pr_{0.95}FeO_{3-\delta}$	1.27	0.034	700	This work
$SrFeO_{3-\delta}$	26.3	-	700	14
$SrFeO_{3-\delta}$	80	0.006	700	15
$SrFe_{0.9}Al_{0.1}O_{3-\delta}$	58	-	700	2
$SrFe_{0.8}Al_{0.2}O_{3-\delta}$	28	-	700	2
$SrFe_{0.8}W_{0.2}O_{3-\delta}$	-	1.16	850	16
$SrFe_{0.75}W_{0.25}O_{3-\delta}$	24		700	15
$SrFe_{0.8}W_{0.1}Ti_{0.1}O_{3-\delta}$	38	-	700	8
$SrFe_{0.85}Zr_{0.15}O_{3-\delta}$	41	0.10	700	1
$SrFe_{0.75}Zr_{0.25}O_{3-\delta}$	8.9	0.1	700	1
$SrFe_{0.9}Si_{0.1}O_{3-\delta}$	35.3	-	700	14
$SrFe_{0.9}Sb_{0.1}O_{3-\delta}$	35	-	700	3
$SrFe_{0.5}Mn_{0.25}Mo_{0.25}O_{3-\delta}$	0.84	6.5	600	6
$Sr_2Fe_{1.5}Mo_{0.5}O_{6-\delta}$		33.9	800	17
$Sr_2Fe_{1.4}Nb_{0.1}Mo_{0.5}O_{6-\delta}$	27.61	15.86	600	18
$Sr_2Fe_{1.4}Ni_{0.1}Mo_{0.5}O_{6-\delta}$	46.3	13.2	600	19,20
$Sr_2TiFe_{0.8}Mo_{0.2}O_{6-\delta}$	0.83	0.87	600	21
$Pr_{0.6}Sr_{0.4}FeO_{3-\delta}$	209	1.3	800	22
$PrBaFe_{1.8}Ta_{0.2}O_5$	20	1.8	700	23
$PrBaFe_{1.9}Cu_{0.1}O_{6-\delta}$	8.5	-	700	13
$PrBaFe_{1.9}Nb_{0.1}O_{5+\delta}$	24.1	2.8	700	24
$PrBa_{0.5}Sr_{0.5}Fe_2O_{5+\delta}$	59	0.09	700	24
$La_{0.8}Sr_{0.2}FeO_{3-\delta}$	122	0.5	700	25
$La_{0.6}Sr_{0.4}Fe_{0.9}Sc_{0.1}O_{3-\delta}$	106	0.3	700	26
$La_{0.3}Sr_{0.7}Fe_{0.9}Ti_{0.1}O_{3-\delta}$	125	0.5	700	27

Table S5. Relative amounts of the different contributions in the O1s and Fe2p core-levels obtained by XPS.

Composition	O1s				Fe2p	
	O_{lat} (%)	O_2^{2-}/O^- (%)	OH^-/O_2 (%)	H_2O (%)	Fe^{3+} (%)	Fe^{4+} (%)
Pr0.8	51.0	16.9	22.9	9.2	71.3	28.7
Pr0.6	46.8	17.9	26.5	8.8	65.9	34.1
Pr0.2	40.1	21.5	28.4	10.0	59.1	40.9

Table S6. Electrode polarization resistance and power density of Fe-based electrodes typically used as symmetrical electrodes.

Electrode Abbreviation	R_p^{air} (Ω cm^2)	$R_p^{H_2}$ (Ω cm^2)	P (mW cm^{-2})	Electrolyte	Ref.
$(Sr_{0.8}Pr_{0.2})_{0.95}FeO_{3-\delta}$-CGO	0.05 $^{700°C}$	0.17 $^{700°C}$		LSGM	This work
$(Sr_{0.6}Pr_{0.4})_{0.95}FeO_{3-\delta}$-CGO	0.04 $^{700°C}$	0.12 $^{700°C}$	630 $^{800°C}$	LSGM	This work
$(Sr_{0.4}Pr_{0.6})_{0.95}FeO_{3-\delta}$-CGO	0.05 $^{700°C}$	0.08 $^{700°C}$		LSGM	This work
$(Sr_{0.2}Pr_{0.8})_{0.95}FeO_{3-\delta}$-CGO	0.09 $^{700°C}$	0.14 $^{700°C}$		LSGM	This work
$Pr_{0.95}FeO_{3-\delta}$-CGO	1.72 $^{700°C}$	-		LSGM	This work
$SrFeO_{3-\delta}$	0.55 $^{700°C}$	-	-	CGO	28
$SrFe_{0.9}Mo_{0.1}O_{3-\delta}$	0.42 $^{700°C}$	-	500 $^{700°C}$	CGO	28
$SrFe_{0.9}Sb_{0.1}O_{3-\delta}$	0.275 $^{700°C}$	-	-	CGO	3
$Sr_2Fe_{1.5}Mo_{0.5}O_{6-\delta}$	0.24 $^{800°C}$	0.27 $^{800°C}$	650 $^{850°C}$	LSGM	29
$Sr_2Fe_{1.5}Mo_{0.5}O_{6-\delta}$-CSO	0.29 $^{800°C}$	0.12 $^{800°C}$	220 $^{800°C}$	LSGM	30
$Sr_2Fe_{1.4}Nb_{0.1}Mo_{0.5}O_{6-\delta}$	0.098 $^{800°C}$	1.5 $^{800°C}$	531$^{800°C}$	LSGM	18
$SrFe_{0.9}Al_{0.1}O_{3-\delta}$	0.15 $^{700°C}$			CSO	2
$SrFe_{0.8}Al_{0.2}O_{3-\delta}$	0.33 $^{700°C}$			CSO	2
$SrFe_{0.8}W_{0.2}O_{3-\delta}$	0.08	0.20 $^{800°C}$	931 $^{850°C}$	LSGM	16
$SrFe_{0.8}W_{0.1}Ti_{0.1}O_{3-\delta}$		1.98 $^{700°C}$	267 $^{800°C}$	YSZ	8
$Sr_{0.9}Fe_{0.8}Sc_{0.1}Co_{0.1}O_{3-\delta}$-CGO	-	0.20	370 $^{800°C}$	YSZ	12
$SrFe_{0.75}Zr_{0.25}O_{3-\delta}$-CGO	0.11$^{700°C}$	0.18 $^{750°C}$	425 $^{800°C}$	LSGM	1
$PrBaFe_2O_{5+\delta}$	0.25 $^{800°C}$	0.48 $^{800°C}$	153 $^{800°C}$	CGO	31
$PrBaFe_{1.9}Cu_{0.1}O_{6-\delta}$	1.7 $^{700°C}$				13
$PrBaFe_{1.8}Ta_{0.2}O_5$	0.17	0.5$^{800°C}$	139 $^{800°C}$	YSZ	23
$La_{0.8}Sr_{0.2}FeO_{3-\delta}$	1.6 $^{700°C}$	5.9 $^{700°C}$	316 $^{800°C}$	YSZ	25
$La_{0.7}Sr_{0.3}Ti_{0.1}Fe_{0.6}Ni_{0.3}O_{3-\delta}$	0.18 $^{700°C}$	0.40 $^{700°C}$	402 $^{800°C}$	LSGM	32
$(La_{0.7}Sr_{0.3})_{0.9}Ti_{0.1}Fe_{0.6}Ni_{0.3}O_{3-\delta}$	0.06 $^{750°C}$	0.21 $^{750°C}$	323 $^{750°C}$	LSGM	33
$La_{0.5}Sr_{0.5}Fe_{0.9}Mo_{0.1}O_{3-\delta}$	0.5 $^{700°C}$	0.28 $^{700°C}$	300 $^{700°C}$	LSGM	34
$La_{0.6}Ca_{0.4}Fe_{0.8}Ni_{0.2}O_{3-\delta}$	0.65 $^{700°C}$		350 $^{800°C}$	CSO	35
$La_{0.6}Ce_{0.1}Sr_{0.3}Fe_{0.95}Ru_{0.05}O_{3-\delta}$-CGO	0.65 $^{700°C}$	0.35 $^{700°C}$	650 $^{700°C}$	LSGM	36

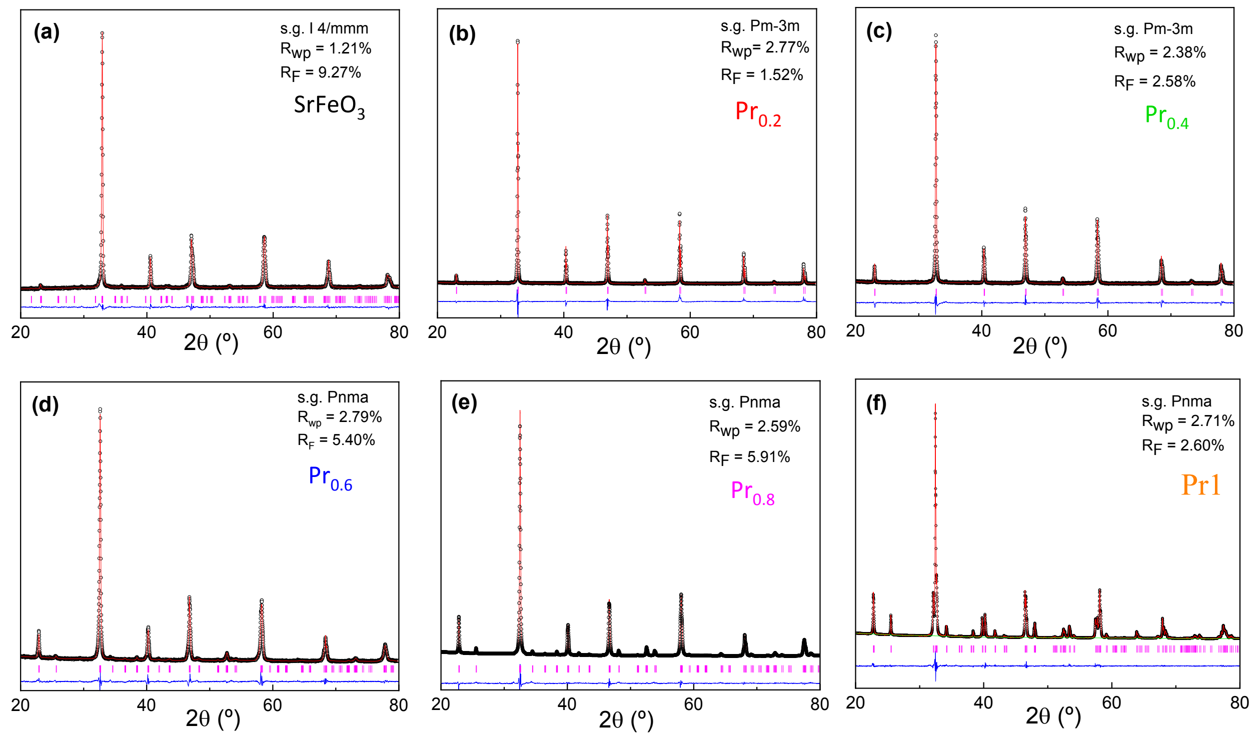

Figure S1. XRPD Rietveld plots of the $Sr_{1-x}Pr_xFeO_{3-\delta}$ (Pr_x) series synthesized in air at 1100 ºC for 1 h: (a) $SrFeO_3$, (b) $Pr_{0.2}$, (c) $Pr_{0.4}$, (d) $Pr_{0.6}$, (e) $Pr_{0.8}$ and (f) Pr_1.

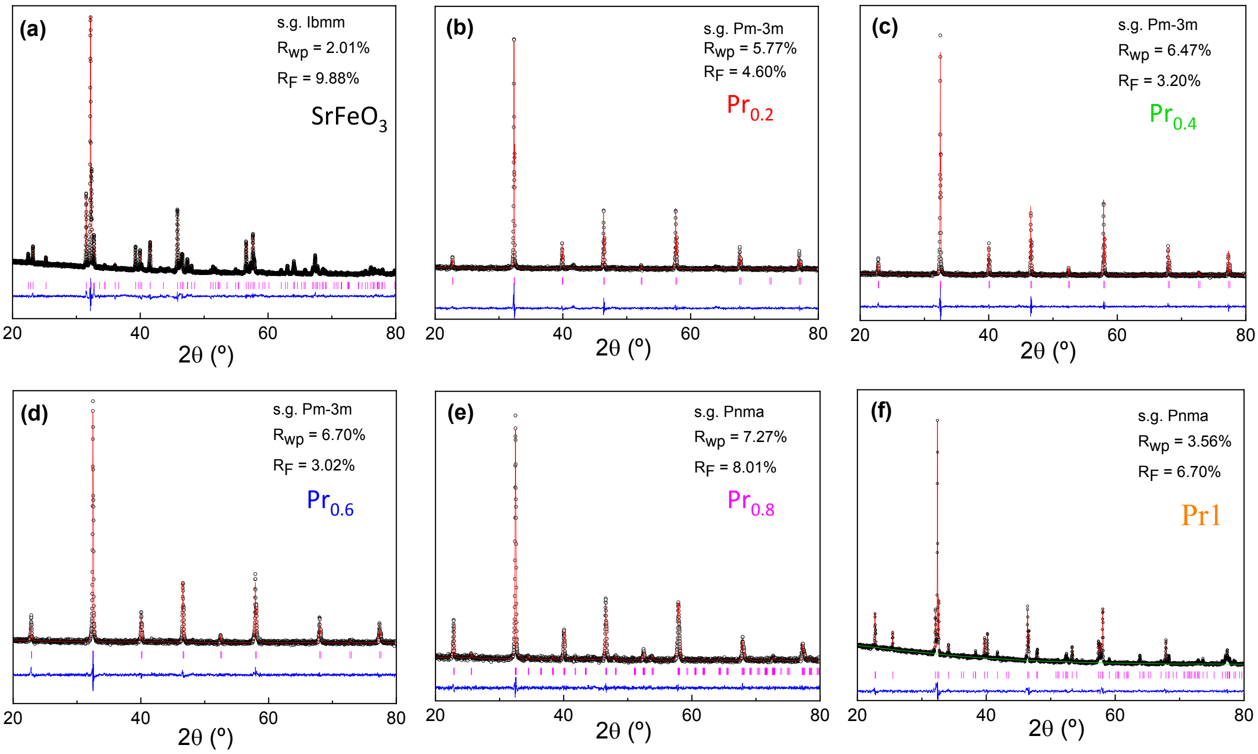

Figure S2. XRPD Rietveld plots of the $Sr_{1-x}Pr_xFeO_{3-\delta}$ (Pr_x) series after annealing at 800 ºC for 24 h in 5% H_2-Ar: (a) $SrFeO_3$, (b) $Pr_{0.2}$, (c) $Pr_{0.4}$, (d) $Pr_{0.6}$, (e) $Pr_{0.8}$ and (f) Pr_1.

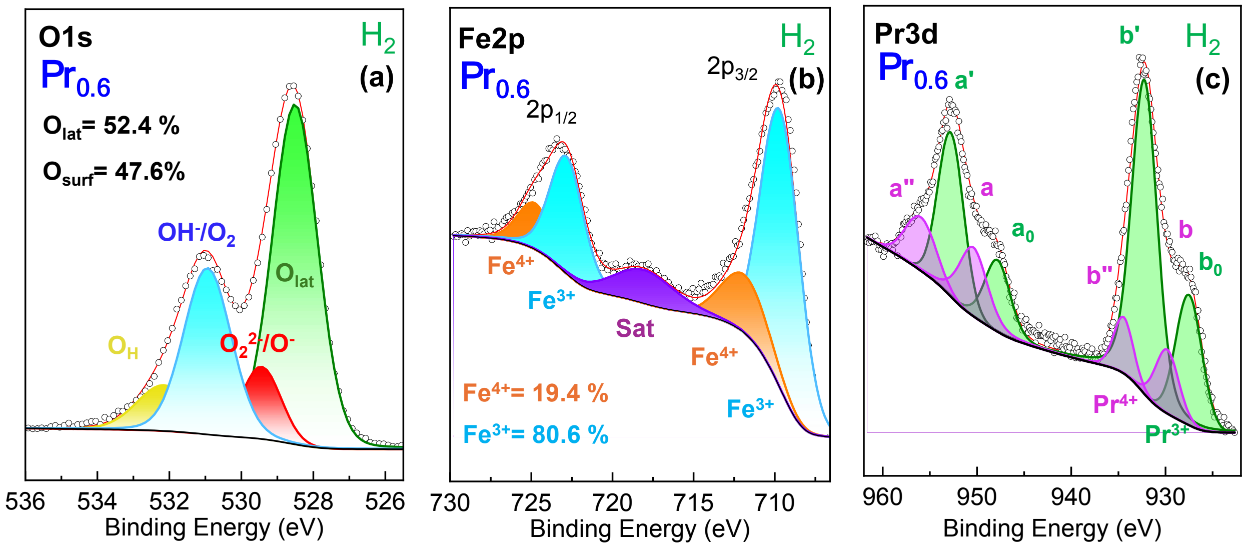

Figure S3. XPS spectra of Pr0.6 after annealing in 5% H_2 for (a) O1s, (b) Fe2p and (c) Pr3d core levels.

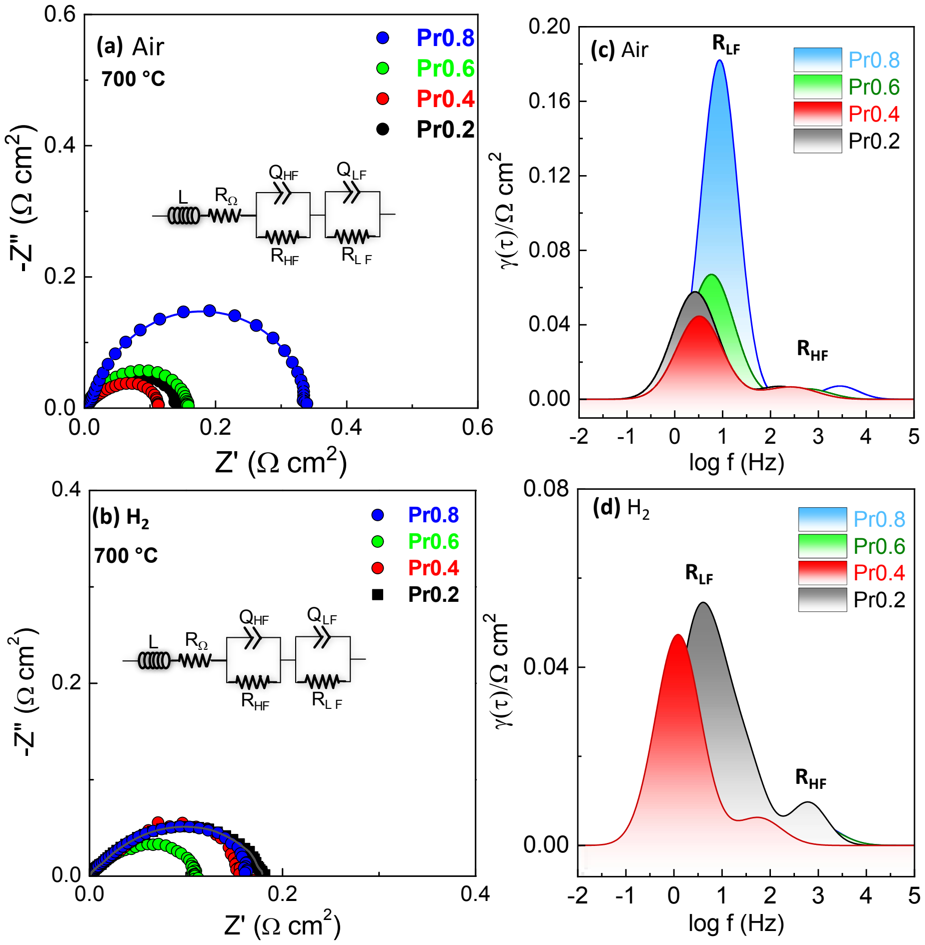

Figure S4. Impedance spectra of $(Sr_{1-x}Pr_x)_{0.95}FeO_{3-\delta}$ electrodes on LSGM electrolyte in (a) air and (b) H_2 atmospheres, along with corresponding DRT curves in (c) air and (d) H_2. The ohmic resistance of the electrolyte has been subtracted for better comparison of the electrode contribution. The equivalent circuit used to fit the data is shown in the insets.

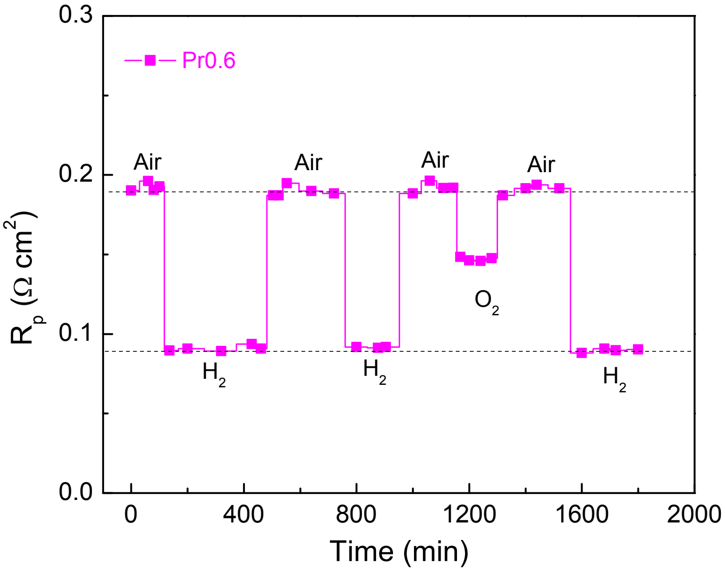

Figure S5. Variation of the polarization resistance after cycling between air and H_2 atmospheres at 700 ºC.

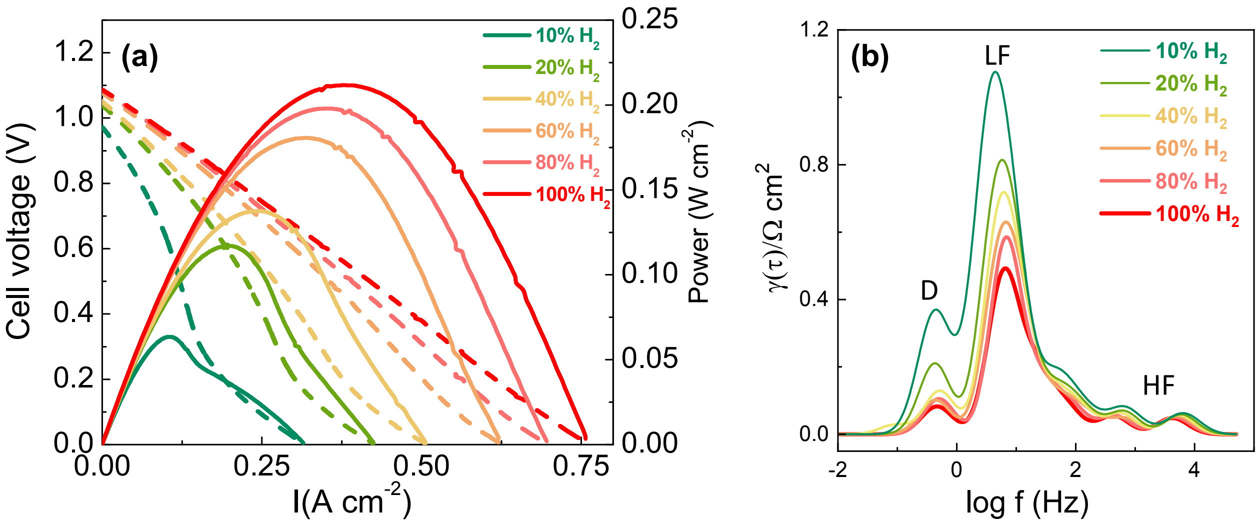

Figure S6. (a) I-V curves measured at 700 ºC with different H_2 fuel concentrations, along with the corresponding (b) DRT curves at OCV.

References

(1) Dos Santos-Gómez, L.; Compana, J. M.; Bruque, S.; Losilla, E. R.; Marrero-López, D. Symmetric Electrodes for Solid Oxide Fuel Cells Based on Zr-Doped $SrFeO_{3-\delta}$. *J Power Sources* **2015**, *279*, 419–427. https://doi.org/10.1016/j.jpowsour.2015.01.043.

(2) Huan, D.; Zhang, L.; Zhu, K.; Li, X.; Zhang, B.; Shi, J.; Peng, R.; Xia, C. Tailoring the Structural Stability, Electrochemical Performance and CO2 Tolerance of Aluminum Doped $SrFeO_3$. *Sep Purif Technol* **2022**, *290*. https://doi.org/10.1016/j.seppur.2022.120843.

(3) Zapata-Ramírez, V.; Mather, G. C.; Pérez-Coll, D. Optimisation of Electrochemical Performance of $Sr(Fe,Sb)O_{3-\delta}$ Air Electrodes for Intermediate-Temperature Solid Oxide Cells through Spray-Pyrolysis Processing. *J Power Sources* **2024**, *600*. https://doi.org/10.1016/j.jpowsour.2024.234243.

(4) Meng, Y.; Sun, L.; Gao, J.; Tan, W.; Chen, C.; Yi, J.; Bouwmeester, H. J. M.; Sun, Z.; Brinkman, K. S. Insights into the CO_2 Stability-Performance Trade-Off of Antimony-Doped $SrFeO_{3-\delta}$ Perovskite Cathode for Solid Oxide Fuel Cells. *ACS Appl Mater Interfaces* **2019**, *11* (12), 11498–11506. https://doi.org/10.1021/acsami.9b00876.

(5) Zapata-Ramírez, V.; Mather, G. C.; Azcondo, M. T.; Amador, U.; Pérez-Coll, D. Electrical and Electrochemical Properties of the $Sr(Fe,Co,Mo)O_{3-\delta}$ System as Air Electrode for Reversible Solid Oxide Cells. *J Power Sources* **2019**, *437*. https://doi.org/10.1016/j.jpowsour.2019.226895.

(6) Zheng, K.; Świerczek, K.; Polfus, J. M.; Sunding, M. F.; Pishahang, M.; Norby, T. Carbon Deposition and Sulfur Poisoning in $SrFe_{0.75}Mo_{0.25}O_{3-\delta}$ and $SrFe_{0.5}Mn_{0.25}Mo_{0.25}O_{3-\delta}$ Electrode Materials for Symmetrical SOFCs. *J Electrochem Soc* **2015**, *162* (9), F1078–F1087. https://doi.org/10.1149/2.0981509jes.

(7) Qiao, J.; Chen, W.; Wang, W.; Wang, Z.; Sun, W.; Zhang, J.; Sun, K. The Ca Element Effect on the Enhancement Performance of $Sr_2Fe_{1.5}Mo_{0.5}O_{6-\delta}$ Perovskite as Cathode for Intermediate-Temperature Solid Oxide Fuel Cells. *J Power Sources* **2016**, *331*, 400–407. https://doi.org/10.1016/j.jpowsour.2016.09.082.

(8) Su, T.; Zhang, T.; Xie, H.; Zhong, J.; Xia, C. Investigation into Structure and Property of W and Ti Co-Doped $SrFeO_3$ Perovskite as Electrode of Symmetrical Solid Oxide Fuel Cell. *Int J Hydrogen Energy* **2022**, *47* (36), 16272–16282. https://doi.org/10.1016/j.ijhydene.2022.03.130.

(9) Yao, C.; Zhang, H.; Dong, Y.; Zhang, R.; Meng, J.; Meng, F. Characterization of Ta/W Co-Doped $SrFeO_{3-\delta}$ Perovskite as Cathode for Solid Oxide Fuel Cells. *J Alloys Compd* **2019**, *797*, 205–212. https://doi.org/10.1016/j.jallcom.2019.05.096.

(10) Zhu, Y.; Sunarso, J.; Zhou, W.; Jiang, S.; Shao, Z. High-Performance $SrNb_{0.1}Co_{0.9-x}Fe_xO_{3-\delta}$ Perovskite Cathodes for Low-Temperature Solid Oxide Fuel Cells. *J Mater Chem A Mater* **2014**, *2* (37), 15454–15462. https://doi.org/10.1039/c4ta03208j.

(11) Shen, L.; Du, Z.; Zhang, Y.; Dong, X.; Zhao, H. Medium-Entropy Perovskites $Sr(Fe_\alpha Ti_\beta Co_\gamma Mn_\zeta)O_{3-\delta}$ as Promising Cathodes for Intermediate Temperature Solid Oxide Fuel Cell. *Appl Catal B* **2021**, *295*. https://doi.org/10.1016/j.apcatb.2021.120264.

(12) Yang, C.; Wang, Y.; Tian, Y.; Wang, Z.; Pu, J.; Ciucci, F.; Chi, B. Electrochemical Performance of Symmetric Solid Oxide Cells Employing a Sc-Doped $SrFeO_{3-\delta}$-Based Electrode. *Chemical Engineering Journal* **2024**, *485*. https://doi.org/10.1016/j.cej.2024.149970.

(13) Matkin, D. E.; Gordeeva, M. A.; Tarutin, A. P.; Medvedev, D. A. $PrBaFe_2O_{6-\delta}$-Based Composites as Promising Electrode Materials for Protonic Ceramic Electrochemical Cells. *J Eur Ceram Soc* **2024**, *44* (10), 5782–5793. https://doi.org/10.1016/j.jeurceramsoc.2024.03.052.

(14) Porras-Vazquez, J. M.; Pike, T.; Hancock, C. A.; Marco, J. F.; Berry, F. J.; Slater, P. R. Investigation into the Effect of Si Doping on the Performance of $SrFeO_{3-\delta}$ SOFC Electrode Materials. *J Mater Chem A Mater* **2013**, *1* (38), 11834–11841. https://doi.org/10.1039/c3ta12113e.

(15) Fernández-Ropero, A. J.; Porras-Vázquez, J. M.; Cabeza, A.; Slater, P. R.; Marrero-López, D.; Losilla, E. R. High Valence Transition Metal Doped Strontium Ferrites for Electrode Materials in Symmetrical SOFCs. *J Power Sources* **2014**, *249*, 405–413. https://doi.org/10.1016/j.jpowsour.2013.10.118.

(16) Cao, Y.; Zhu, Z.; Zhao, Y.; Zhao, W.; Wei, Z.; Liu, T. Development of Tungsten Stabilized $SrFe_{0.8}W_{0.2}O_{3-\delta}$ Material as Novel Symmetrical Electrode for Solid Oxide Fuel Cells. *J Power Sources* **2020**, *455*. https://doi.org/10.1016/j.jpowsour.2020.227951.

(17) Xu, Z.; Hu, X.; Wan, Y.; Xue, S.; Zhang, S.; Zhang, L.; Zhang, B.; Xia, C. Electrochemical Performance and Anode Reaction Process for Ca Doped $Sr_2Fe_{1.5}Mo_{0.5}O_{6-\delta}$ as Electrodes for Symmetrical Solid Oxide Fuel Cells. *Electrochim Acta* **2020**, *341*. https://doi.org/10.1016/j.electacta.2020.136067.

(18) Gou, M.; Ren, R.; Sun, W.; Xu, C.; Meng, X.; Wang, Z.; Qiao, J.; Sun, K. Nb-Doped $Sr_2Fe_{1.5}Mo_{0.5}O_{6-\delta}$ Electrode with Enhanced Stability and Electrochemical Performance for Symmetrical Solid Oxide Fuel Cells. *Ceram Int* **2019**, *45* (12), 15696–15704. https://doi.org/10.1016/j.ceramint.2019.03.130.

(19) Dai, N.; Feng, J.; Wang, Z.; Jiang, T.; Sun, W.; Qiao, J.; Sun, K. Synthesis and Characterization of B-Site Ni-Doped Perovskites $Sr_2Fe_{1.5-x}Ni_xMo_{0.5}O_{6-\delta}$ (x = 0, 0.05, 0.1, 0.2, 0.4) as Cathodes for SOFCs. *J Mater Chem A Mater* **2013**, *1* (45), 14147–14153. https://doi.org/10.1039/c3ta13607h.

(20) Feng, J.; Yang, G.; Dai, N.; Wang, Z.; Sun, W.; Rooney, D.; Qiao, J.; Sun, K. Investigation into the Effect of Fe-Site Substitution on the Performance of $Sr_2Fe_{1.5}Mo_{0.5}O_{6-\delta}$ Anodes for SOFCs. *J Mater Chem A Mater* **2014**, *2* (41), 17628–17634. https://doi.org/10.1039/c4ta03216k.

(21) Niu, B.; Jin, F.; Zhang, L.; Shen, P.; He, T. Performance of Double Perovskite Symmetrical Electrode Materials $Sr_2TiFe_{1-x}Mo_xO_{6-\delta}$ (x = 0.1, 0.2) for Solid Oxide Fuel Cells. *Electrochim Acta* **2018**, *263*, 217–227. https://doi.org/10.1016/j.electacta.2018.01.062.

(22) Admasu Beshiwork, B.; Sirak Teketel, B.; Luo, X.; Tian, D.; Yang, Q.; Zhu, S.; Chen, Y.; Timurkutluk, B.; Lin, B. Nanoengineering Electrode for Yttria-Stabilized Zirconia-Based Symmetrical Solid Oxide Fuel Cells to Achieve Superior Output Performance. *Sep Purif Technol* **2022**, *295*. https://doi.org/10.1016/j.seppur.2022.121174.

(23) Liu, C.; Wang, F.; Ni, Y.; Wang, S.; Qian, B.; Ni, Q.; Zheng, Y.; Chen, H.; Ge, L. Ta-Doped $PrBaFe_2O_{5+\delta}$ Double Perovskite as a High-Performance Electrode Material for Symmetrical Solid Oxide Fuel Cells. *Int J Hydrogen Energy* **2023**, *48* (26), 9812–9822. https://doi.org/10.1016/j.ijhydene.2022.11.237.

(24) Wang, F.; Qi, J.; Shan, P.; Qian, B.; Xie, L.; Zheng, Y.; Chen, H.; Ge, L. Co-Free and Sr-Free Double-Perovskite Oxide $PrBaFe_{1.9}Nb_{0.1}O_{5+\delta}$ as a Potential Electrode Material for Symmetrical Solid Oxide Fuel Cells. *Ionics (Kiel)* **2024**. https://doi.org/10.1007/s11581-024-05724-w.

(25) Tian, D.; Lin, B.; Yang, Y.; Chen, Y.; Lu, X.; Wang, Z.; Liu, W.; Traversa, E. Enhanced Performance of Symmetrical Solid Oxide Fuel Cells Using a Doped Ceria Buffer Layer. *Electrochim Acta* **2016**, *208*, 318–324. https://doi.org/10.1016/j.electacta.2016.04.189.

(26) Liu, X.; Han, D.; Zhou, Y.; Meng, X.; Wu, H.; Li, J.; Zeng, F.; Zhan, Z. Sc-Substituted $La_{0.6}Sr_{0.4}FeO_{3-\delta}$ Mixed Conducting Oxides as Promising Electrodes for Symmetrical Solid Oxide Fuel Cells. *J Power Sources* **2014**, *246*, 457–463. https://doi.org/10.1016/j.jpowsour.2013.07.111.

(27) Canales-Vázquez, J.; Ruiz-Morales, J. C.; Marrero-López, D.; Peña-Martínez, J.; Núñez, P.; Gómez-Romero, P. Fe-Substituted (La,Sr)TiO_3 as Potential Electrodes for Symmetrical Fuel Cells (SFCs). *J Power Sources* **2007**, *171* (2), 552–557. https://doi.org/10.1016/j.jpowsour.2007.05.094.

(28) Zapata-Ramírez, V.; Rosendo-Santos, P.; Amador, U.; Ritter, C.; Mather, G. C.; Pérez-Coll, D. Optimisation of High-Performance, Cobalt-Free $SrFe_{1-x}Mo_xO_{3-\delta}$ Cathodes for Solid Oxide Fuel Cells Prepared by Spray Pyrolysis. *Renew Energy* **2022**, *185*, 1167–1176. https://doi.org/10.1016/j.renene.2021.12.121.

(29) Liu, Q.; Dong, X.; Xiao, G.; Zhao, F.; Chen, F. A Novel Electrode Material for Symmetrical SOFCs. *Advanced Materials* **2010**, *22* (48), 5478–5482. https://doi.org/10.1002/adma.201001044.

(30) Osinkin, D. A.; Kolchugin, A. A.; Bogdanovich, N. M.; Beresnev, S. M. Performance and Redox Stability of a Double–Layer $Sr_2Fe_{1.5}Mo_{0.5}O_{6-\delta}$ – Based Electrode for Solid State Electrochemical Application. *Electrochim Acta* **2020**, *361*. https://doi.org/10.1016/j.electacta.2020.137058.

(31) Qi, J.; Liu, C.; Li, S.; Xie, L.; Chen, H.; Ge, L.; Zheng, Y. Enhancing the Catalytic Activity of $PrBaFe_2O_{5+\delta}$ Double Perovskite with $BaCoO_{3-\delta}$ Modification as an Electrode Material for Symmetrical Solid Oxide Fuel Cells. *Int J Hydrogen Energy* **2024**, *71*, 259–267. https://doi.org/10.1016/j.ijhydene.2024.05.267.

(32) Hanif, M. B.; Gao, J. T.; Shaheen, K.; Wang, Y. P.; Yasir, M.; Zhang, S. L.; Li, C. J.; Li, C. X. Performance Evaluation of Highly Active and Novel $La_{0.7}Sr_{0.3}Ti_{0.1}Fe_{0.6}Ni_{0.3}O_{3-\delta}$ Material Both as Cathode and Anode for Intermediate-Temperature Symmetrical Solid Oxide Fuel Cell. *J Power Sources* **2020**, *472*. https://doi.org/10.1016/j.jpowsour.2020.228498.

(33) Hanif, M. B.; Gao, J. T.; Shaheen, K.; Wang, Y. P.; Yasir, M.; Li, C. J.; Li, C. X. Highly Active and Novel A-Site Deficient Symmetric Electrode Material $(Sr_{0.3}La_{0.7})_{1-x}(Fe_{0.7}Ti_{0.3})_{0.9}Ni_{0.1}O_{3-\delta}$ and Its Effect on Electrochemical Performance of SOFCs. *Int J Hydrogen Energy* **2021**, *46* (12), 8778–8791. https://doi.org/10.1016/j.ijhydene.2020.12.093.

(34) Bian, L.; Liu, C.; Li, S.; Peng, J.; Li, X.; Guan, L.; Liu, Y.; Peng, J. H.; An, S.; Song, X. Highly Stable $La_{0.5}Sr_{0.5}Fe_{0.9}Mo_{0.1}O_{3-\delta}$ Electrode for Reversible Symmetric Solid Oxide Cells. *Int J Hydrogen Energy* **2020**, *45* (38), 19813–19822. https://doi.org/10.1016/j.ijhydene.2020.05.117.

(35) Yang, G.; Su, C.; Chen, Y.; Tadé, M. O.; Shao, Z. Nano $La_{0.6}Ca_{0.4}Fe_{0.8}Ni_{0.2}O_{3-\delta}$ Decorated Porous Doped Ceria as a Novel Cobalt-Free Electrode for "Symmetrical" Solid Oxide Fuel Cells. *J Mater Chem A Mater* **2014**, *2* (45), 19526–19535. https://doi.org/10.1039/c4ta03485f.

(36) Wang, J.; Fu, L.; Yang, J.; Wu, K.; Zhou, J.; Wu, K. Cerium and Ruthenium Co-Doped $La_{0.7}Sr_{0.3}FeO_{3-\delta}$ as a High-Efficiency Electrode for Symmetrical Solid Oxide Fuel Cell. *Journal of Rare Earths* **2021**, *39* (9), 1095–1099. https://doi.org/10.1016/j.jre.2021.01.009.
